# Composition–Structure–Property Relationships in PLA-nHA-Mg Hybrid Biocomposites: Effect of Ceramic–Metallic Reinforcement Ratio on Mechanical and Physicochemical Performance

**DOI:** 10.3390/polym18192438

**Published:** 2026-10-07

**Authors:** K. Hariharan, B. Veera Siva Reddy, Dola Sundeep, C. Chandrasekhara Sastry, Robert Cep

**Affiliations:** 1 Department of Mechanical Engineering, Dr Mahalingam College of Engineering and Technology, Coimbatore 642003, Tamil Nadu, India; 2 Department of Mechanical Engineering, Indian Institute of Information Technology Design and Manufacturing Kurnool (IIITDM Kurnool), Kurnool 518008, Andhra Pradesh, India; 3 School of Electrical and Computer Sciences, Indian Institute of Technology Bhubaneswar (IIT BBS), Argul, Jatni, Khordha 752050, Odisha, India; 4 Department of Machining, Assembly and Engineering Metrology, Faculty of Mechanical Engineering, VSB-Technical University of Ostrava, 70800 Ostrava, Czech Republic

**Keywords:** poly(lactic acid), nano-hydroxyapatite, magnesium, hybrid biocomposite, mechanical performance, composition–structure–property relationship

## Abstract

Poly(lactic acid) (PLA) hybridized with nano-hydroxyapatite (nHA) and elemental magnesium (Mg) offers a route to tune load-bearing behavior while retaining a biodegradable polymer matrix. This study compares neat PLA, binary PLA-10Mg and PLA-10nHA controls, and three ternary hybrids containing a constant 10 wt.% total reinforcement at Mg:nHA ratios of 1:1, 3:1, and 1:3. Tensile, compressive, and three-point flexural responses were interpreted together with differential scanning calorimetry (DSC), X-ray diffraction (XRD), and Fourier-transform infrared (FTIR) spectroscopy. Ten specimens were tested per formulation in each mechanical assay (five initial specimens and five confirmatory specimens), and the mechanical data were consolidated across all ten specimens and are reported as an arithmetic mean ± standard deviation (SD). The recorded response was loading-mode-dependent: PLA-5Mg-5nHA combined a tensile stress of 52.0 ± 1.56 MPa with the largest tensile peak displacement ratio of 23.42 ± 0.70%; PLA-7.5Mg-2.5nHA showed the largest flexural peak load of 490.4 ± 14.71 N; and PLA-2.5Mg-7.5nHA combined 52.8 ± 1.58 MPa tensile stress, 93.7 ± 2.81 MPa compressive stress, and 431.5 ± 12.95 N flexural peak load. DSC showed a high-temperature heat-flow event at 365.6–372.6 °C under the stated mixed-purge conditions; this is treated as an operational thermal marker and not a melting or decomposition temperature. XRD remained matrix-dominated with reinforcement-sensitive features, while FTIR showed composition-dependent shifts in the C-O/C-O-C region. The results indicate that Mg:nHA partitioning alters the recorded loading-mode response within the tested 10 wt.% reinforcement window, while particle-scale mechanisms and biological performance require further validation.

## 1. Introduction

Large and critical-sized bone defects remain difficult to treat because the native regenerative capacity of bone becomes inadequate when the defect size, vascularity, trauma severity, or pathological conditions exceed the biological healing window. Conventional grafting approaches can restore structure, but donor-site morbidity, limited autograft availability, disease-transmission concerns, and variable integration continue to motivate synthetic, degradable alternatives. Modern bone-repair materials are therefore expected to do more than occupy a defect: they must provide sufficient temporary mechanical support, interact favorably with the mineralizing tissue environment, and degrade without producing an adverse local response. Hydroxyapatite (HAp)-based biomaterials remain central to this strategy because their calcium-phosphate chemistry and osteoconductive surface resemble the mineral component of bone [1,2,3].

Poly(lactic acid) (PLA) is one of the most established biodegradable polymer families considered for tissue-engineering scaffolds, fixation concepts, and additively manufactured biomedical structures. Its appeal derives from its processability, degradability, and useful strength-to-density ratio. Nevertheless, unmodified PLA is not an ideal bone analog. It is comparatively bioinert at the surface and can display brittle or notch-sensitive failure depending on its molecular weight and processing history, while its hydrolytic degradation can alter the local chemical environment. Reviews of PLA/HAp systems consequently emphasize the need to couple the polymer with an inorganic phase when simultaneous mechanical support and bone-facing surface chemistry are required [2,4,5,6].

Nano-hydroxyapatite (nHA) is an especially attractive ceramic reinforcement because nano-scale particles offer high interfacial area and chemistry close to the apatite fraction of mineralized tissue. PLA/nHA research has shown, however, that the mechanical response is strongly concentration- and processing-dependent. Wang et al. reported that adding a very high 50 wt.% nHA fraction to 3D-printed PLA reduced the compressive strength from 35.41 to 17.80 MPa while improving osteogenic functionality, highlighting a trade-off between the bioactive phase content and structural performance [7]. In contrast, Injorhor et al. found a non-monotonic tensile response in PLA/nHAp films, where the tensile strength increased from 38.21 MPa for PLA to 66.41 MPa at 5 phr nHAp but decreased to 45.80 MPa at 10 phr, which they associated with filler agglomeration above an optimum loading [8]. Studies of PLA/PCL/nHA systems have similarly shown that the particle fraction can alter crystallization, dispersion, and tensile/flexural behavior instead of acting as a simple linear stiffener [3,9,10,11,12,13].

Magnesium introduces a different design function. Mg is a lightweight biodegradable metal of interest for orthopedic and vascular applications, as it gradually dissolves in physiological environments and participates in normal biological processes. Its principal challenge is that degradation can be too rapid if the surface and microstructural conditions are not controlled [14]. In polymer-based hybrids, Mg can provide a metallic load-transfer phase and a source of Mg-containing degradation products, while the polymer can partially isolate the metal from direct fluid exposure. This makes Mg fundamentally different from an inert ceramic filler and motivates investigation of polymer–ceramic–metal combinations rather than binary composites alone [15,16,17,18,19].

Several studies have already explored Mg-containing HAp/PLA systems, but the chemical form of Mg differs from that in the present work. Swetha et al. used Mg-doped nHAp as a coating on 3D PLA/gelatin scaffolds and demonstrated osteogenic effects [20]. Xu et al. incorporated nHA and nano-MgO into 3D-printed PLA; the addition of nHA increased the yield stress from about 18 to 22 MPa, and the subsequent addition of nMgO increased it to about 24 MPa while also modifying crystallization and degradation behavior [21]. Hajibeygi and Darvishi developed PLA/HAp nanocomposites containing modified Mg(OH)_2_ and showed that combined fillers could alter tensile strength and thermal stability [22]. More recent Mg-Zn-substituted HAp/PLA hybrid systems have focused on ionic substitution, interfacial chemistry, cytocompatibility, corrosion resistance, and implant coatings [23,24]. These studies establish the value of Mg-bearing phases but do not directly resolve the behavior of a PLA matrix reinforced simultaneously by discrete nHA ceramic and elemental Mg particles [15,16,17,18].

A second unresolved issue is reinforcement partitioning. Hybrid composites are often described in terms of their total filler content, but the relative fraction of metallic and ceramic reinforcement can govern load transfer, interfacial debonding, crack deflection, crystallization, and local chemistry in different ways. The recent PLA/HA/TiO_2_ literature has explicitly shown that changing the secondary reinforcement ratio can modify mechanical, morphological, and thermal properties [25]. Likewise, Mg/HAp-based metal-matrix systems demonstrate that hybrid reinforcement effects need not be additive, because the reinforcements interact through microstructure and interface formation [26]. Accordingly, a polymer–ceramic–metal system requires a composition-resolved analysis rather than a simple comparison between reinforced and unreinforced PLA [10,11,12,16].

The specific research gap addressed here is therefore the lack of an integrated, composition-controlled assessment of PLA containing nHA and elemental Mg as physically discrete reinforcements, with the Mg:nHA ratio systematically varied and evaluated across multiple loading modes together with thermal and structural characterizations. The existing literature is dominated by PLA/nHA binary composites, Mg-doped apatite, MgO or Mg(OH)_2_ additions, or Mg-based metallic matrices. Consequently, the extent to which elemental Mg:nHA partitioning affects tensile, compressive, and flexural performance in a PLA-based matrix remains insufficiently defined, particularly when correlated with DSC, XRD, and FTIR evidence [3,15,16,17,18]. The present study addresses this gap using neat PLA and binary PLA/Mg and PLA/nHA controls, together with three hybrid compositions containing 90 wt.% PLA and 10 wt.% combined Mg + nHA at Mg:nHA ratios of 5:5, 7.5:2.5, and 2.5:7.5. The central hypothesis is that the mechanical response will be non-monotonic, because Mg and nHA influence deformation and interfacial constraints differently, and that the most favorable formulation will depend on the loading mode rather than on a single strength metric. Tensile, compressive, and three-point flexural responses are therefore evaluated alongside DSC, XRD, and FTIR. The work is intentionally framed as a composition–structure–property study: biological potential is motivated by the constituent selection, whereas direct bioactivity, degradation, and cellular behavior are not claimed without dedicated experiments.

## 2. Materials and Methods

### 2.1. Materials and Procurement Quality Control

Commercially procured medical-grade poly(lactic acid) (PLA) resin was used as the continuous biodegradable polymer matrix. The PLA resin was supplied by Formlabs. Commercial nano-hydroxyapatite (nHA) powder and elemental magnesium (Mg) powder, each specified at a nominal purity of ≥99%, were used as the ceramic and metallic reinforcing phases, respectively; both were procured from the same source [2,3,15].

To reduce moisture- and contamination-related variability, PLA, nHA, and Mg were stored in sealed containers under dry laboratory conditions and handled using clean, dry tools. Constituent masses were measured gravimetrically using an analytical balance, and all compositions were calculated on a total composite mass basis and are reported in weight percent (wt.%). The same weighing and handling procedures were applied to each batch. A constant total reinforcement fraction of 10 wt.% was adopted for all reinforced compositions so that differences in the measured response could be interpreted primarily in terms of reinforcement chemistry and Mg:nHA partitioning rather than changes in total filler loading.

For reporting consistency with accepted biomaterials practice, the nHA phase is described using the hydroxyapatite nomenclature Ca_10_(PO_4_)_6_(OH)_2_, with an ideal stoichiometric Ca/P atomic ratio of approximately 1.67 [1,3].

### 2.2. Composite Formulations and Experimental Design

Six material systems were investigated using a composition-controlled design that comprised one neat-polymer reference, two binary controls, and three ternary hybrid composites, as indicated in Table 1. Neat PLA (S1) served as the matrix reference. The binary controls PLA-10Mg (S2) and PLA-10nHA (S3) contained 90 wt.% PLA and 10 wt.% of a single reinforcement. For the ternary hybrids, the PLA fraction was held at 90 wt.% and the total reinforcement fraction at 10 wt.%, while the relative Mg:nHA ratio was systematically varied. PLA-5Mg-5nHA (S4) represented the balanced 1:1 condition, PLA-7.5Mg-2.5nHA (S5) as the Mg-rich 3:1 condition, and PLA-2.5Mg-7.5nHA (S6) as the nHA-rich 1:3 condition.

This design provides three direct levels of comparison: (i) the effect of adding reinforcement relative to neat PLA; (ii) the effect of reinforcement chemistry through direct comparison of PLA-10Mg and PLA-10nHA; and (iii) the effect of Mg:nHA partitioning at a fixed total reinforcement content. Fixing the total filler content in S2–S6 minimizes a major compositional confounder and strengthens subsequent composition–structure–property comparisons across the mechanical, thermal and spectroscopic datasets.

### 2.3. Specimen Preparation, Test-Standard Framework and Quality Assurance

**Figure 1:**
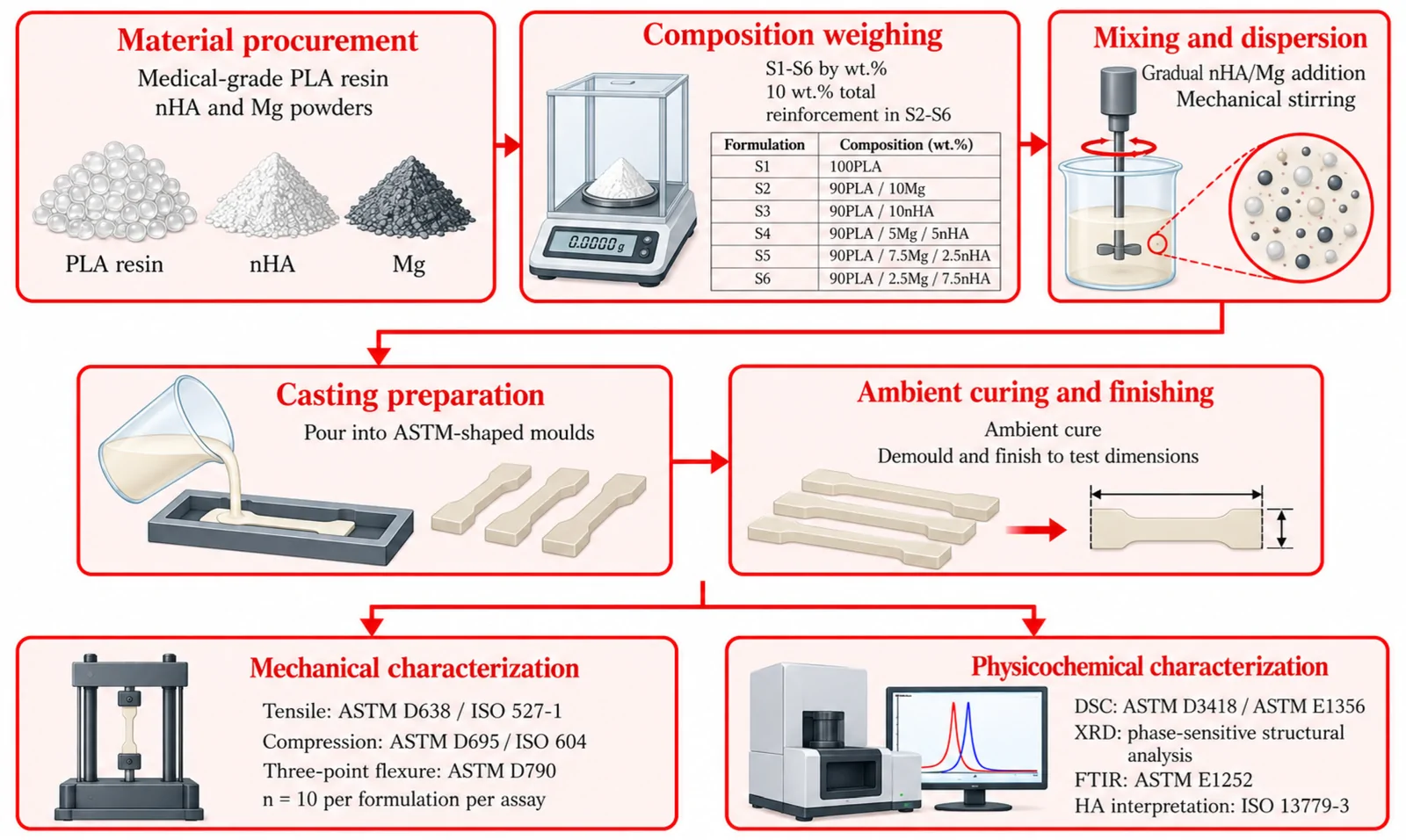
Experimental workflow for preparation and characterization of the PLA-nHA-Mg biocomposites.

The six formulations were manufactured by the casting route recorded for this study. The PLA resin, nHA powder, and Mg powder were weighed according to the compositions in Table 1. The required nHA and/or Mg powder was introduced gradually into the PLA matrix while mechanical stirring was applied to promote a uniform dispersion. The resulting mixture was then poured into ASTM-shaped molds, cured at an ambient laboratory temperature, demolded, and finished to the required dimensions. The same preparation sequence was applied to all six compositions to limit process-induced variability. For manuscript reporting, tensile testing was referenced to ASTM D638-22 [27], with ISO 527-1:2019 [28] as supplementary guidance; compression testing was referenced to ASTM D695-26 [29] and ISO 604:2002 [30]; and three-point flexural testing was referenced to ASTM D790-25 [31]. DSC reporting used ASTM D3418-21 [32] and ASTM E1356-25 [33]; FTIR acquisition and qualitative interpretation followed ASTM E1252-98(2021) [34]; and hydroxyapatite phase interpretation was referenced to ISO 13779-3:2018 [35]. These standards are used as test/reporting frameworks rather than as a claim of strict formal compliance where archived dimensional details are incomplete. The complete sequence from material procurement and composition control through to casting, specimen preparation, mechanical testing, and physicochemical characterization is summarized in Figure 1.

The archived machine reports retain the specimen length, machine-entered cross-sectional area, load-cell capacity and crosshead speed, but they do not preserve every dimensional parameter needed to independently demonstrate strict conformance with the current editions of each standard. Accordingly, the mechanical results are reported using the instrument-recorded engineering quantities and the raw load–displacement/stress–strain records without retroactively reconstructing unavailable dimensions. In particular, flexural stress is not independently recalculated when the support span and individual specimen widths/thicknesses are absent from the archived record. This reporting approach preserves data traceability and avoids introducing geometric assumptions that are not supported by the source files.

Good data-integrity practice was maintained throughout the post-processing workflow. Raw instrument exports were retained unchanged; no smoothing was applied to mechanical, DSC, XRD, or FTIR datasets used for quantitative interpretation; sample codes were cross-mapped before analysis; machine acceptance/rejection flags were preserved; and derived plots were regenerated from the numerical exports rather than instrument screenshots. Any departure from complete standard traceability is stated explicitly instead of being treated as full formal compliance.

### 2.4. Mechanical Testing

Mechanical characterization of the PLA-nHA-Mg composites was carried out using a PC2000 electronic tensometer/Mini-UTM (Kudale Instruments Pvt. Ltd. (KIPL), India) equipped with a nominal 20,042 N load cell. Tensile, compressive, and three-point flexural tests were used to compare the load-bearing responses of the six formulations. For each formulation and each loading mode, n = 10 specimens were tested under the same nominal settings, comprising five specimens in the initial series and five confirmatory specimens. For each specimen, the corresponding peak machine-derived response was extracted from the test record. The ten specimen-level values for each formulation and loading mode were then consolidated by calculating the arithmetic mean and sample standard deviation (SD), and all tabulated mechanical results are therefore reported as mean ± SD (n = 10). The figures show singular unsmoothed test traces for visualization of the characteristic curve shape, whereas quantitative comparisons are based on the n = 10 consolidated statistics reported in Table 2.

#### 2.4.1. Tensile Testing

Tensile testing was performed in accordance with the ASTM D638-22 framework [27] for polymeric materials. The specimens were tested at a crosshead speed of 5 mm min^−1^, with a nominal specimen length of 50 mm. The cross-sectional area entered into the testing system was 90 mm^2^ for S1–S4 and 75 mm^2^ for S5–S6. During testing, load, displacement, engineering stress, and engineering strain were recorded continuously until specimen failure. The resulting engineering stress–strain curves were used to determine the tensile response of each composition, including the peak tensile load, peak tensile stress, and corresponding displacement. The recorded data were analyzed in their original numerical forms without curve smoothing or artificial modification.

#### 2.4.2. Compression Testing

Compression testing was conducted with reference to the ASTM D695-26 [29] and ISO 604:2002 [30] procedures for rigid polymeric materials. Tests were performed at a constant crosshead speed of 3 mm min^−1^. The nominal specimen dimension recorded in the test system was 2.5 mm, with a corresponding cross-sectional area of 31.185 mm^2^. Load and displacement were continuously acquired during compression, and the corresponding engineering stress response was obtained from the test-system output. The compression behavior of the different compositions was evaluated in terms of the peak compressive load, peak engineering stress, and associated deformation. The complete experimental data series was retained for comparative analysis of reinforcement-dependent mechanical response.

#### 2.4.3. Three-Point Flexural Testing

Flexural behavior was evaluated using a three-point bending configuration in accordance with the ASTM D790-25 [31] test framework. The specimens had a nominal recorded length of 60 mm and were tested at a crosshead speed of 3 mm min^−1^. The cross-sectional areas entered into the testing system were 63.5 mm^2^ for S1 and S2 and 76.2 mm^2^ for S3–S6. The applied load and corresponding displacement were continuously recorded throughout the test. Flexural performance was assessed using the experimentally measured load–displacement response together with the nominal engineering stress reported by the testing system. Peak flexural load, peak displacement, and the corresponding engineering stress were extracted for comparison among the neat PLA, binary composites, and Mg-nHA hybrid formulations.

#### 2.4.4. Mechanical Data Analysis

The mechanical responses of the six compositions were evaluated from the tensile, compression, and flexural test records. For each formulation and assay, n = 10 specimens were tested, comprising five initial specimens and five confirmatory specimens under the same nominal test settings. Either the peak stress or peak load and the corresponding displacement parameter were extracted for each individual specimen. For each formulation and loading mode, the ten specimen-level peak values were consolidated as the arithmetic mean and sample standard deviation (SD); thus, the values reported in Table 2 are the mean ± SD of n = 10. The initial and confirmatory series were pooled because they were obtained under the same formulation-specific test conditions. Across the accepted mechanical metrics, the reported SDs correspond to approximately 2.9–3.0% of their respective group means, indicating close clustering of the repeated measurements. Acceptance or rejection followed the acquisition-system status retained in the test records; the S3 tensile result remained classified as rejected and is reported only for traceability, not for tensile-performance ranking. Relative changes in the mechanical parameters were calculated from the group means with respect to neat PLA (S1) using the equation below. No ANOVA or pairwise inferential significance test was applied; percentage differences and rankings are therefore interpreted as descriptive comparisons of the consolidated group means.

$$\Delta P(\%)=\frac{P_{i}-P_{\mathrm{PLA}}}{P_{\mathrm{PLA}}}\times 100$$

where P_i_ represents the mechanical parameter of the reinforced composition and P_PLA_ represents the corresponding value for neat PLA. This approach was used consistently to evaluate the effects of reinforcement chemistry and the Mg:nHA ratio on tensile, compressive, and flexural performance.

### 2.5. Differential Scanning Calorimetry

DSC was performed using a NETZSCH DSC 3500 Sirius (NETZSCH GmbH, Germany). The archived dataset contained one heating scan per formulation (n = 1 analytical measurement per composition). The recorded sample masses were 3.2 mg (S1), 3.4 mg (S2), 3.3 mg (S3), 3.1 mg (S4), 2.5 mg (S5), and 3.4 mg (S6). Heating traces were acquired from approximately 20 to 500 °C at 10 K min^−1^ using Concavus aluminum pans with pierced lids. The instrument method recorded the protective nitrogen at 50 mL min^−1^, purge gas 1 oxygen at 40 mL min^−1^, and purge gas 2 nitrogen at 60 mL min^−1^. A separate neat-PLA cooling segment from 500 to 25 °C at 10 K min^−1^ was also retained. The instrument convention defined the exothermic heat flow as positive. ASTM D3418-21 [32] and ASTM E1356-25 [33] were used as reporting frameworks; the single-run values are therefore interpreted descriptively and are not assigned formulation-level uncertainty.

The conventional melting range of PLA is generally reported at approximately 150–180 °C, with values near 150–153 °C also reported for PLA/Mg systems [5,18,19]. Therefore, the pronounced event observed here at approximately 320–400 °C is not assigned as the PLA melting temperature. Because the archived DSC method extends to 500 °C and includes an oxygen purge stream, the feature is described here strictly as an operational high-temperature heat-flow event under the stated mixed-purge conditions. Its peak temperature was obtained from the minimum heat-flow value in this interval and is used only for relative comparison among compositions. The signal may include overlapping thermo-oxidative, volatilization, chain-scission, and heat-capacity contributions [5,18,19].

A lower-temperature feature near 100–105 °C was also examined. The software-annotated PLA + nHA trace identified an onset at 101.7 °C, a midpoint at 102.9 °C, and an endpoint at 104.1 °C. In the present analysis, this response is discussed as a low-amplitude transition/relaxation feature, because its absolute assignment is sensitive to the polymer grade and prior thermal history [5,18].

### 2.6. X-Ray Diffraction

XRD measurements were performed using a Bruker D8 Advance theta/theta diffractometer (Bruker, USA) with Cu Kα radiation (λ1 = 1.5406 Å), operated at 40 kV and 30 mA. One archived diffraction pattern per formulation was available (n = 1 analytical measurement per composition). Patterns were recorded over 2θ = 10–80° using the archived step size of 0.020439°. Raw intensity-versus-angle data were used for peak-position analysis. For comparative presentation only, individual patterns were normalized independently and vertically offset to improve visibility; normalization was not used for quantitative phase-fraction analysis. The archive did not contain an independent repeat pattern for S5, so the unusually weak S5 diffraction contrast cannot be evaluated for reproducibility in the present dataset.

Phase interpretation was conducted conservatively with reference to accepted hydroxyapatite characterization principles, including ISO 13779-3:2018 [35]. Because separate reference scans of the as-received nHA and Mg powders and internal standards were not included in the archive, overlapping reflections were not deconvoluted into quantitative phase fractions, and crystallinity was not inferred from normalized plots alone. Accordingly, the XRD results are used to identify qualitative changes in pattern features and reinforcement-associated reflections rather than to claim quantitative phase purity.

### 2.7. Fourier-Transform Infrared Spectroscopy

FTIR spectra were acquired using a JASCO FTIR X-series spectrometer (JASCO Systems, USA) over approximately 400–4000 cm^−1^ at 4 cm^−1^ spectral resolution. One archived spectrum per formulation was available (n = 1 analytical measurement per composition); each spectrum comprised 16 instrumental accumulations, which improve signal averaging but do not constitute independent specimen replicates. Spectra were acquired in transmittance mode at an automatic scan speed of 2 mm s^−1^. Data acquisition and qualitative interpretation followed ASTM E1252-98(2021) [34]. Raw spectra were used to determine principal band positions. For stacked visualization only, each spectrum was normalized independently so that band-position changes could be compared without implying absolute transmittance equivalence. Interpretation focused on the PLA ester-carbonyl region (~1700–1780 cm^−1^), C-H stretching region (~2800–3000 cm^−1^), C-O/C-O-C regions (~1200–1300 and 1020–1160 cm^−1^), and phosphate-sensitive regions near ~960–980 and 500–620 cm^−1^. Band shifts and intensity changes were treated as evidence of changes in the local chemical environment or reinforcement contribution; the formation of new covalent bonds was not claimed solely from FTIR data.

### 2.8. Replication and Statistical Scope

The replication level differed between the mechanical and physicochemical measurements. Each mechanical loading mode used n = 10 specimens per formulation, comprising five initial specimens and five confirmatory specimens tested under the same nominal conditions. The ten mechanical replicate values were pooled for each formulation and loading mode and are reported as a consolidated arithmetic mean ± sample SD. The SD therefore quantifies within-formulation repeatability across the ten tested specimens; for the accepted mechanical metrics, the SD was approximately 2.9–3.0% of the corresponding mean. No inferential ANOVA or pairwise significance testing was performed, so comparisons among formulations are based on the group means and their observed within-group dispersion. In contrast, the physicochemical dataset contained one DSC heating scan, one XRD pattern, and one FTIR spectrum per formulation; the 16 FTIR accumulations are repeated acquisitions within a single spectrum rather than independent specimens. Accordingly, no SD, confidence interval, or inferential statistical comparison is assigned to the DSC, XRD, or FTIR values.

## 3. Results and Discussion

The results are discussed as a coupled composition–structure–property dataset rather than as independent mechanical, thermal, and spectroscopic measurements. Because the total reinforcement fraction was fixed at 10 wt.% for S2–S6, the observed differences are associated with reinforcement chemistry and Mg:nHA partitioning at a common total filler content. The mechanical trends can therefore be discussed in relation to plausible effects of metallic load redistribution, ceramic constraints, and particle–matrix interactions; however, these mechanisms are interpretations of the macroscopic and physicochemical data rather than direct microstructural observations.

### 3.1. Mechanical Response

The mechanical response varied strongly with reinforcement chemistry and loading mode. The formulation showing the highest tensile response was not the same as that showing the highest compressive or flexural response, indicating that Mg and nHA influence the macroscopic response differently. Tension is particularly sensitive to interfacial load transfer and crack opening; compression emphasizes matrix confinement and localized deformation; and flexure combines tensile, compressive, and shear components through the specimen thickness. These loading-mode differences provide a basis for mechanistic interpretation, but they do not, by themselves, identify the local deformation mechanism. Unless otherwise stated, all mechanical peak values discussed in Section 3.1.1, Section 3.1.2, Section 3.1.3 and Section 3.1.4 are consolidated arithmetic mean ± SD values for n = 10 specimens; single curves shown in the figures are used only to illustrate response shape, while Table 2 provides a statistical summary.

#### 3.1.1. Tensile Behavior

**Figure 2:**
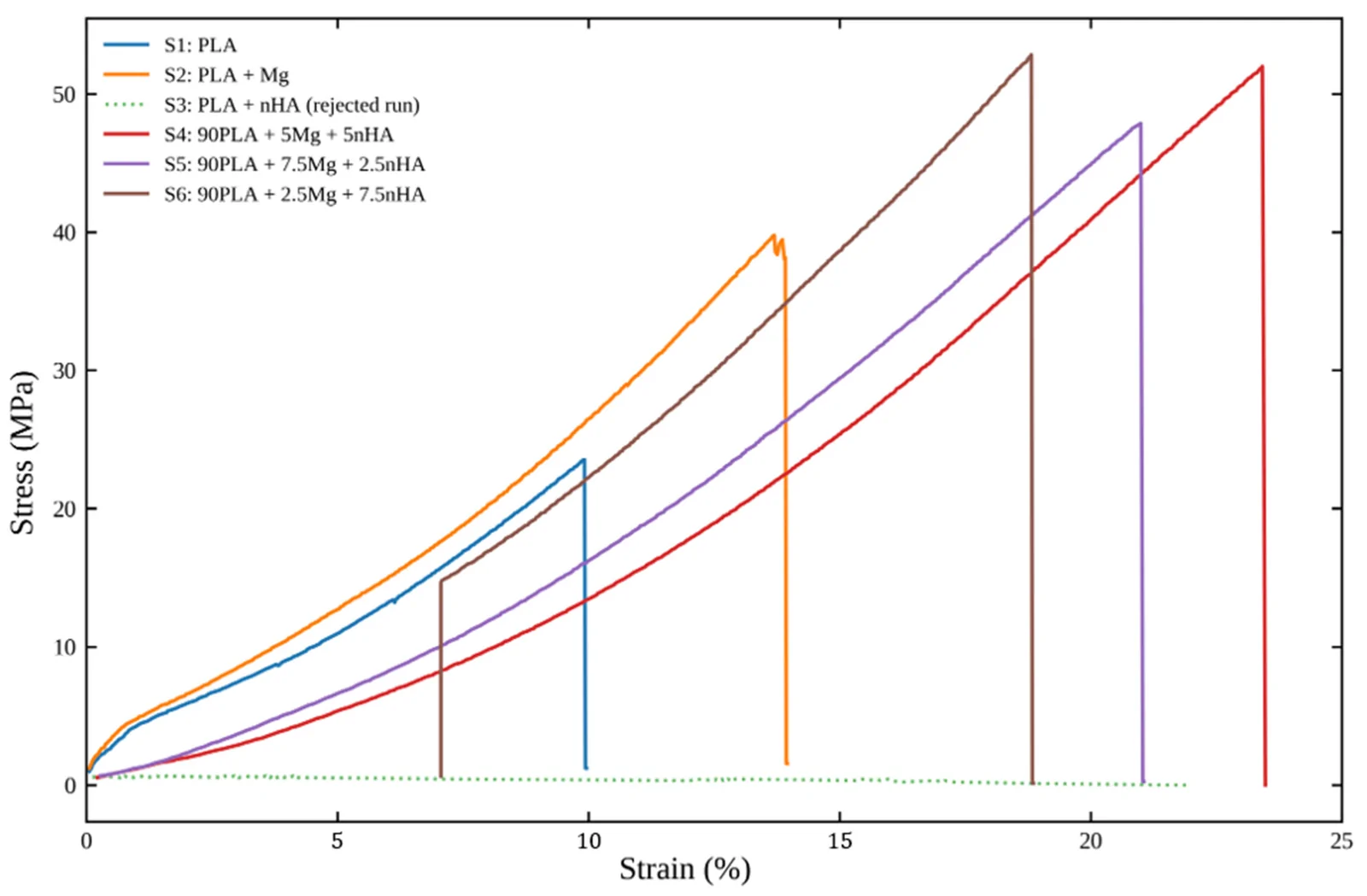
Tensile stress–strain responses of neat PLA and PLA-Mg-nHA composites (x-axis: strain; y-axis: stress). One unsmoothed test trace per formulation is shown to illustrate the characteristic response shape; quantitative tensile results are reported as a consolidated mean ± SD (n = 10) in Table 2. The S3 trace is retained for traceability but excluded from quantitative tensile interpretation because it was rejected by the acquisition system.

**Figure 3:**
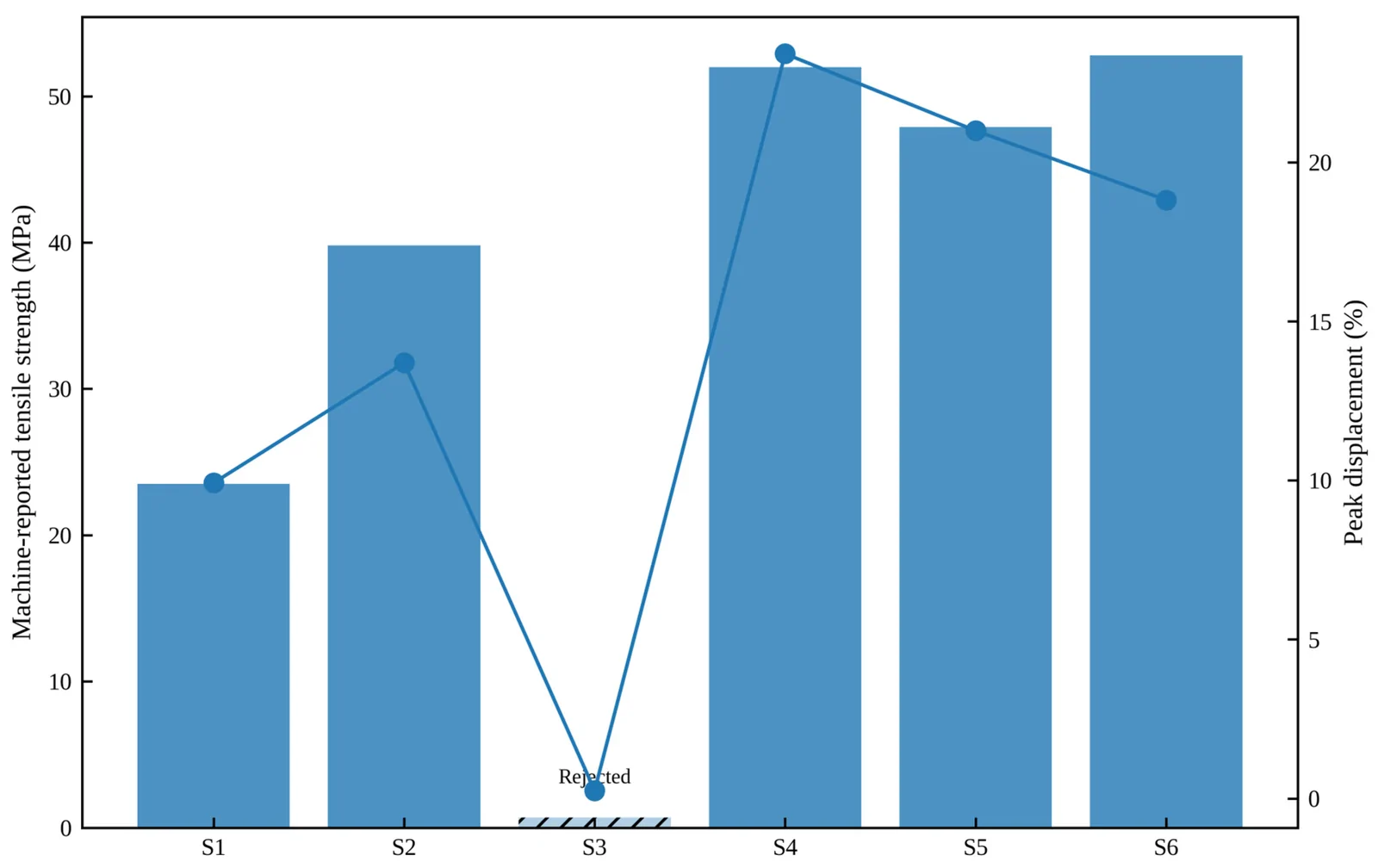
Group-mean peak engineering tensile stress and corresponding peak displacement ratios for S1–S6. The associated SD values are reported in Table 2. The S3 tensile result is retained only for traceability and is not included in composition ranking.

The engineering tensile stress–strain responses are presented in Figure 2, while Figure 3 summarizes the corresponding peak engineering stress and peak displacement ratio. Neat PLA (S1) reached 23.5 ± 0.70 MPa at a peak displacement ratio of 9.92 ± 0.30%. Incorporation of 10 wt.% Mg in S2 increased the mean tensile stress to 39.8 ± 1.19 MPa, corresponding to an improvement of 69.4% relative to S1, while the peak displacement ratio increased to 13.70 ± 0.41%. The accepted ternary hybrids exhibited even greater tensile responses: S4, S5, and S6 reached 52.0 ± 1.56, 47.9 ± 1.44, and 52.8 ± 1.58 MPa, respectively, corresponding to increases of 121.3, 103.8, and 124.7% relative to neat PLA. Their peak displacement ratios were 23.42 ± 0.70, 21.00 ± 0.63, and 18.82 ± 0.56%. The mean tensile stress of 23.5 MPa measured for the cast neat-PLA reference is lower than the approximately 50–65 MPa values commonly quoted for dense injection-molded PLA. The comparison is not directly like-for-like because the present specimens were produced by the study’s casting/ambient-cure route rather than by pressure-assisted injection molding. The tensile response of PLA is sensitive to the commercial grade and its molecular characteristics, moisture and conditioning, thermal and processing history, degree of consolidation, residual porosity, surface conditions, and defect population [5,8,36]. In particular, a cast specimen can exhibit a lower apparent tensile strength than a well-consolidated injection-molded specimen when pressure-assisted densification is absent and casting-related heterogeneity is present. The S1 mean is therefore used as the process-matched internal baseline for the present composition series and should not be interpreted as the universal intrinsic tensile strength of PLA.

The increase from 23.5 ± 0.70 MPa in S1 to 39.8 ± 1.19 MPa in S2 is consistent with an Mg-associated change in the tensile load-bearing response instead of Mg acting solely as an inert inclusion. A plausible interpretation is that part of the applied load is transferred between the continuous polymer matrix and the dispersed metallic phase through the particle–matrix interface. Because Mg has stiffness and deformation characteristics different from both PLA and nHA, its presence may alter the local stress field and the onset of strain localization [15,17,19]. Direct interfacial stress transfer was not measured in the present study, so this explanation is treated as a mechanistic hypothesis rather than a directly observed process.

The higher tensile stresses measured for S4–S6 are consistent with a combined influence of the Mg and nHA phases on the tensile response. nHA can provide a relatively rigid particulate constraint, while Mg introduces a mechanically dissimilar metallic phase. The resulting macroscopic behavior may therefore reflect the combined effects of matrix continuity, particle distribution, and particle–matrix interactions rather than a simple linear sum of filler strengths [10,11,12,13,15]. This interpretation remains provisional because particle dispersion and interfacial morphology were not directly imaged.

S4 is particularly informative because it combines a tensile stress of 52.0 ± 1.56 MPa with the largest peak displacement ratio of 23.42 ± 0.70%. This simultaneous increase in stress-bearing capacity and deformation suggests that the 1:1 Mg:nHA ratio is associated with a favorable tensile strength–deformation balance within the present composition set. One possible explanation is that a heterogeneous distribution of local stiffnesses spreads deformation over a larger matrix volume. In contrast, S6 reaches the highest accepted mean tensile stress, 52.8 ± 1.58 MPa, at a lower peak displacement ratio of 18.82 ± 0.56%. The nHA-rich condition is therefore consistent with greater particulate constraint, although direct evidence of the local constraint mechanism would require microscopy or other interfacial characterizations [11,12,15].

The Mg-rich S5 reaches 47.9 ± 1.44 MPa, lower than S4 and S6 despite the same 10 wt.% total reinforcement. This non-monotonic response shows that filler quantity alone does not account for the observed tensile trend and suggests a role for reinforcement partitioning and particle–matrix interactions. As the Mg fraction increases, changes in particle spacing, local stiffness mismatch, or interfacial deformation could contribute to the response. Such processes are plausible explanations based on composite mechanics, but localized shear, debonding, or particle rearrangement was not directly observed in the present experiments [10,11,12,15,19].

This behavior agrees with the composition-sensitive response reported for PLA/nHA systems. Injorhor et al. observed an increase in tensile strength from 38.21 MPa for neat PLA to 66.41 MPa at 5 phr nHAp, followed by a reduction to 45.80 MPa at 10 phr, showing that reinforcement efficiency reflects competition between load transfer and filler-induced heterogeneity [8]. PLA/PCL/nHA composites likewise exhibit particle-fraction-dependent changes in their tensile and flexural responses [9], while modified HAp/Mg-containing PLA systems demonstrate that combined fillers can alter both mechanical response and matrix mobility [22]. The present ternary system follows the same general principle but extends it to discrete elemental Mg and nHA phases.

The consolidated S3 tensile result was 0.7 ± 0.02 MPa with a peak displacement ratio of 0.24 ± 0.01%, and the acquisition-system status classified the tensile result as rejected. It is therefore retained in Figure 2 and Figure 3 and Table 2 only for traceability and is excluded from tensile-performance ranking and mechanistic interpretation.

#### 3.1.2. Compression Behavior

**Figure 4:**
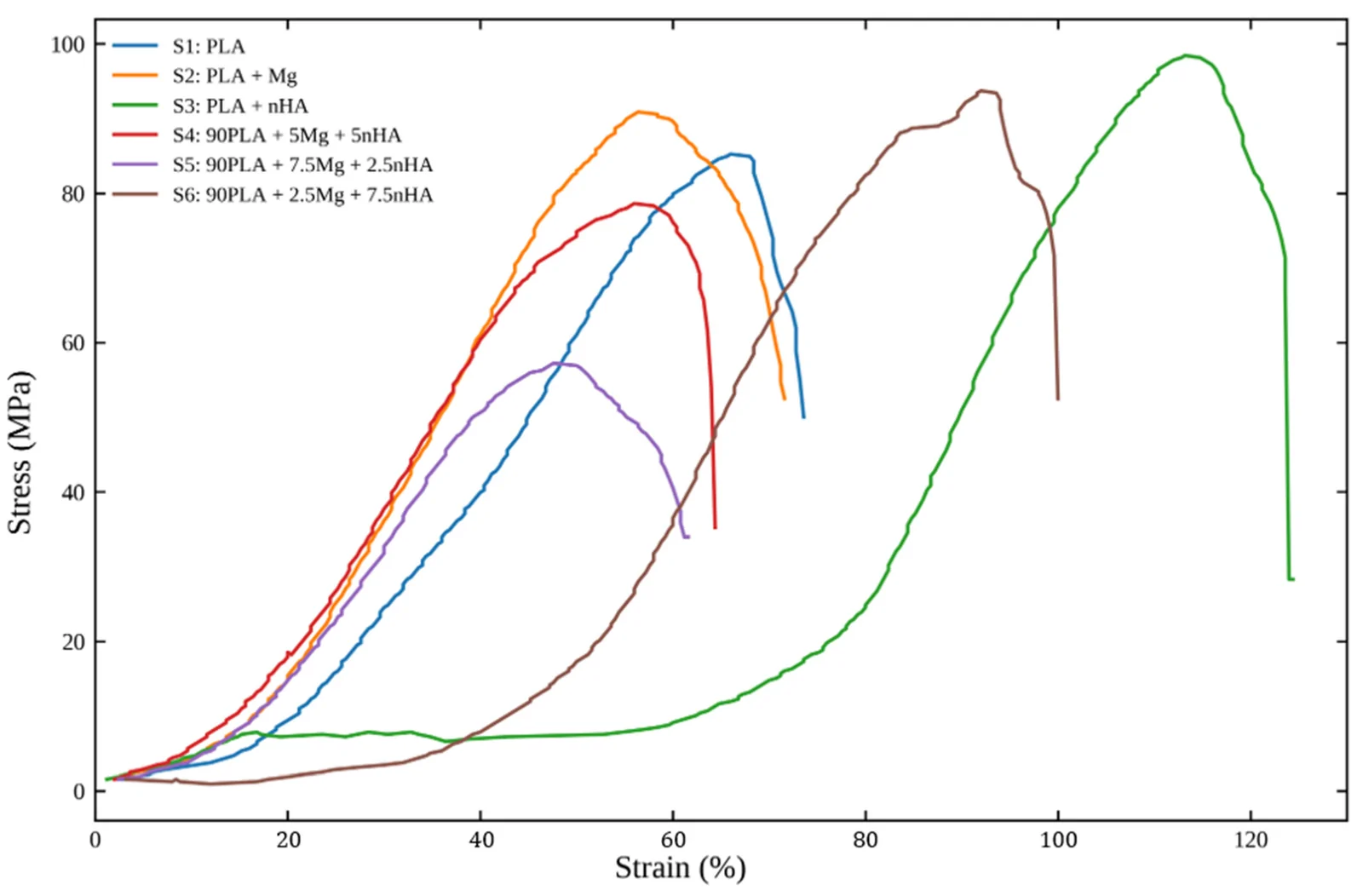
Compression stress–strain responses of S1–S6 (x-axis: strain; y-axis: stress). One unsmoothed test trace per formulation is shown for response-shape visualization; quantitative compression results are reported as consolidated mean ± SD (n = 10).

**Figure 5:**
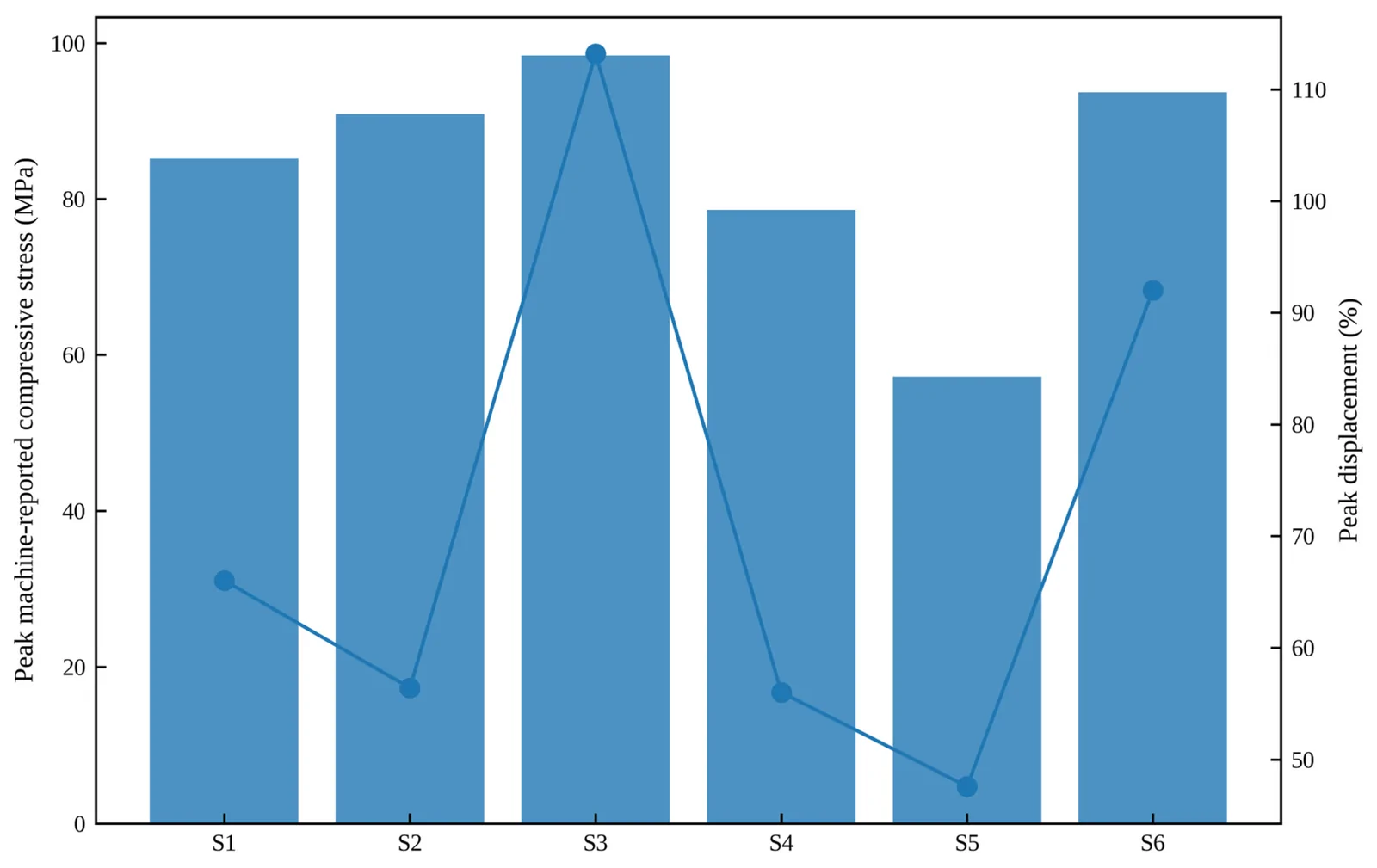
Group-mean peak engineering compression stress and the corresponding peak displacement ratios for the six formulations. The associated SD values are reported in Table 2.

The compression responses are shown in Figure 4 and Figure 5. Neat PLA reached a peak engineering stress of 85.2 ± 2.56 MPa. S2 increased this value to 90.9 ± 2.73 MPa (+6.7%), whereas the binary nHA composite, S3, reached 98.4 ± 2.95 MPa (+15.5%), the highest mean value in the compression dataset. Among the ternary hybrids, S6 retained a high stress of 93.7 ± 2.81 MPa (+10.0% relative to S1), while S4 decreased to 78.6 ± 2.36 MPa (−7.7%) and the Mg-rich S5 decreased to 57.2 ± 1.72 MPa (−32.9%). The corresponding peak displacement ratios were 66.0 ± 1.98, 56.4 ± 1.69, 113.2 ± 3.40, 56.0 ± 1.68, 47.6 ± 1.43, and 92.0 ± 2.76% for S1–S6. Compression generally suppresses crack opening while emphasizing matrix confinement, localized deformation, particle contact, and shear. These expected composite mechanical effects provide a plausible framework for interpreting the comparatively high S3 and S6 responses, but the present bulk of the tests do not directly observe local triaxiality, particle-induced constraint, or shear localization. These mechanisms are therefore treated as qualitative interpretations rather than as direct evidence [10,12,13].

The large displacement ratios recorded for S3 and S6 are consistent with progressive deformation under compression rather than immediate tensile-type fracture. Under compression, defects that would open in tension may instead close, allowing continued load-bearing as the polymer and particles deform. The high deformation of the nHA-containing compositions may therefore reflect matrix confinement and progressive load redistribution [10,12,13].

The Mg-rich S5 shows the opposite macroscopic response. Its peak stress decreases to 57.2 ± 1.72 MPa and the peak displacement ratio to 47.6 ± 1.43%. Because S2–S6 all contain 10 wt.% total reinforcement, the difference cannot be attributed to total filler content alone. It may instead be associated with the redistribution of reinforcement from a predominantly ceramic to a more metallic particle population. Under compression, such a change could alter local strain heterogeneity, matrix flow, or interfacial shear; these possibilities are proposed to rationalize the measured trend and are not direct observations [15,17,19].

The increase from 57.2 ± 1.72 MPa for S5 to 93.7 ± 2.81 MPa for S6 as the Mg:nHA ratio changes from 3:1 to 1:3 is therefore a clear composition-dependent trend with a constant total filler content. The result is consistent with a stronger constraining contribution from the higher nHA fraction, but it does not uniquely establish the local deformation mechanism. Xu et al. similarly showed that nHA and a Mg-bearing phase can cooperate to modify the compressive response of PLA-based scaffolds [21], while the broader PLA hybrid literature shows a strong dependence on reinforcement ratios and processing architectures [7,25].

#### 3.1.3. Three-Point Flexural Behavior

**Figure 6:**
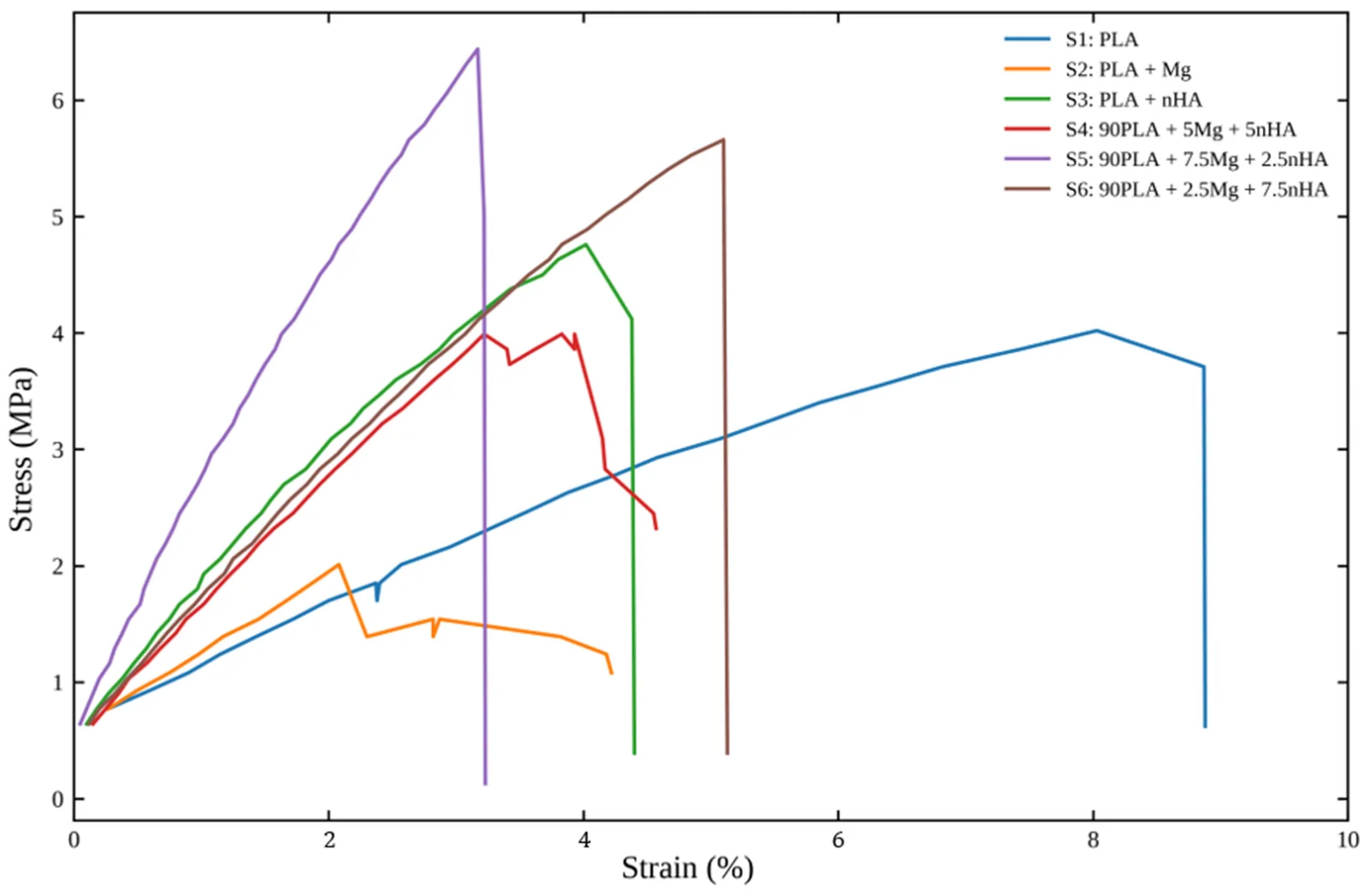
Three-point flexural stress–strain responses of S1–S6 (x-axis: strain; y-axis: stress). One unsmoothed test trace per formulation is shown for curve-shape visualization; quantitative flexural results are consolidated across n = 10 specimens and reported as mean ± SD.

**Figure 7:**
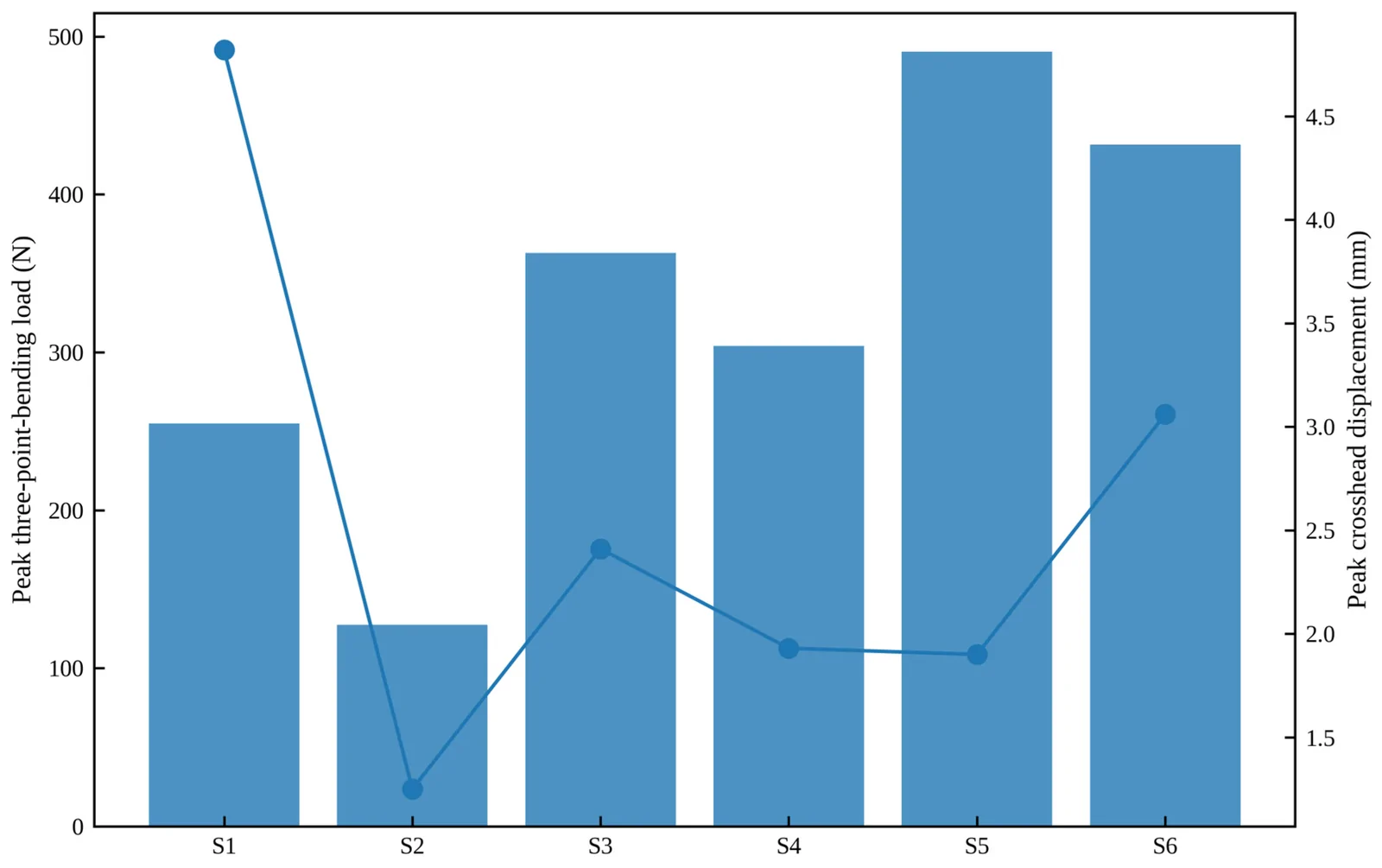
Peak three-point-bending load and the corresponding crosshead displacement from the plotted test traces. S5 exhibits the highest peak load response, whereas S6 combines a high peak load with greater displacement at the peak response. Consolidated peak load means ± SD (n = 10).

The three-point flexural response is presented in Figure 6 and Figure 7. Neat PLA carried a peak load of 255.0 ± 7.65 N. S2 carried only 127.5 ± 3.82 N, representing a 50.0% reduction relative to S1, whereas S3 increased to 362.9 ± 10.89 N (+42.3%). The ternary hybrids carried 304.0 ± 9.12 N for S4, 490.4 ± 14.71 N for S5, and 431.5 ± 12.95 N for S6, corresponding to changes of +19.2, +92.3, and +69.2%, respectively. The mean nominal flexural stresses followed the same ordering: 4.0 ± 0.12, 2.0 ± 0.06, 4.8 ± 0.14, 4.0 ± 0.12, 6.4 ± 0.19, and 5.7 ± 0.17 MPa for S1–S6. The original five-specimen series was supplemented by five confirmatory specimens for each composition under the same flexural conditions, giving n = 10 per formulation. All ten specimen-level peak values were pooled to obtain the mean ± SD reported in Table 2. The comparatively small SDs indicate close repeatability of the formulation-level peak response, while the single unsmoothed curves in Figure 6 are retained only to show the characteristic load-deformation shape and local curve features.

Flexural loading imposes a through-thickness stress gradient, with one surface predominantly in tension and the opposite surface predominantly in compression. The measured response can therefore involve a combination of tensile crack initiation, compressive deformation, and interfacial shear. This mixed stress state may help explain why the flexural ranking differs from the uniaxial tensile and compression rankings, but the present tests do not directly resolve the local failure sequence. The poor flexural response of S2 despite its improved tensile response illustrates the sensitivity of the material to loading path. A metallic particle that contributes to axial load-bearing in tension may act differently under bending, where the outer surface is more sensitive to local defects and mixed normal/shear stresses. The higher flexural load of S3 (362.9 ± 10.89 N, approximately 1.42 times the neat-PLA mean value) is consistent with a possible contribution from nHA-induced stiffness and crack-path perturbation. However, crack deflection, particle bridging, and interfacial integrity were not directly observed in this study; these mechanisms are therefore discussed as plausible interpretations supported by the prior literature [9,10,11,12,13,37].

The most pronounced flexural response occurs for S5, which supports a mean peak load of 490.4 ± 14.71 N, almost twice the mean peak load of neat PLA. This is notable because S5 also shows the lowest mean compressive stress. The contrast suggests that the Mg-rich composition responds favorably to the non-uniform stress field in bending, but it does not by itself identify the underlying micromechanism. A possible explanation is that the Mg-rich phase changes the local compliance and stress redistribution while the smaller nHA fraction contributes a rigid constraint; these effects may act together under flexure, although direct particle-scale evidence is not available [15,16,19].

In the plotted flexural traces, S5 reaches its peak load at approximately 1.90 mm, whereas the S6 trace reaches its peak at approximately 3.06 mm. The consolidated S6 peak load is 431.5 ± 12.95 N. S5 therefore represents a high-load, lower-displacement flexural state, while S6 combines a high load with greater displacement accommodation. The difference is consistent with composition-dependent changes in stiffness, crack initiation, and progressive damage evolution, but the present macroscopic data cannot distinguish the individual contributions of these mechanisms.

The reversal between S5 under compression and bending is an important macroscopic result of the study. It shows that hybrid reinforcement produces loading-mode-dependent behavior rather than a universal strengthening effect. The result is compatible with changes in stress-transfer pathways and phase interactions reported for PLA-based hybrid composites and HAp-containing Mg-based systems [22,25,26].

#### 3.1.4. Cross-Mode Mechanical Response

The combined mechanical dataset shows that the Mg:nHA ratio is associated with loading-mode-specific performance. S2 illustrates the effect of introducing Mg without nHA: the mean tensile stress rises by 69.4% and the mean compressive stress by 6.7%, while the mean flexural peak load falls by 50.0%. These macroscopic changes are consistent with different roles of the metallic phase under axial and bending loads, but they should not be interpreted as direct proof of a particular interfacial load-transfer mechanism. S4 occupies a different mechanical regime. It combines 52.0 ± 1.56 MPa tensile stress with the largest tensile peak displacement ratio (23.42 ± 0.70%), while its compressive and flexural responses remain moderate. The 1:1 Mg:nHA partition is therefore associated with a greater tensile deformation capacity within the present dataset. S5 is flexure-dominant: its tensile response remains high at 47.9 ± 1.44 MPa and its flexural peak load reaches 490.4 ± 14.71 N, but its compressive stress falls to 57.2 ± 1.72 MPa. In contrast, S6 provides a broader combination of responses, with 52.8 ± 1.58 MPa tensile stress, 93.7 ± 2.81 MPa compressive stress and 431.5 ± 12.95 N flexural peak load.

The SD values in Table 2 quantify within-formulation dispersion across the ten mechanical specimens. For the accepted mechanical metrics, the SDs are approximately 2.9–3.0% of the corresponding means, supporting close repeatability of the measured peak responses. No ANOVA or pairwise inferential significance testing was applied; formulation comparisons and percentage changes are therefore based on the consolidated group means and are interpreted descriptively.

**Figure 8:**
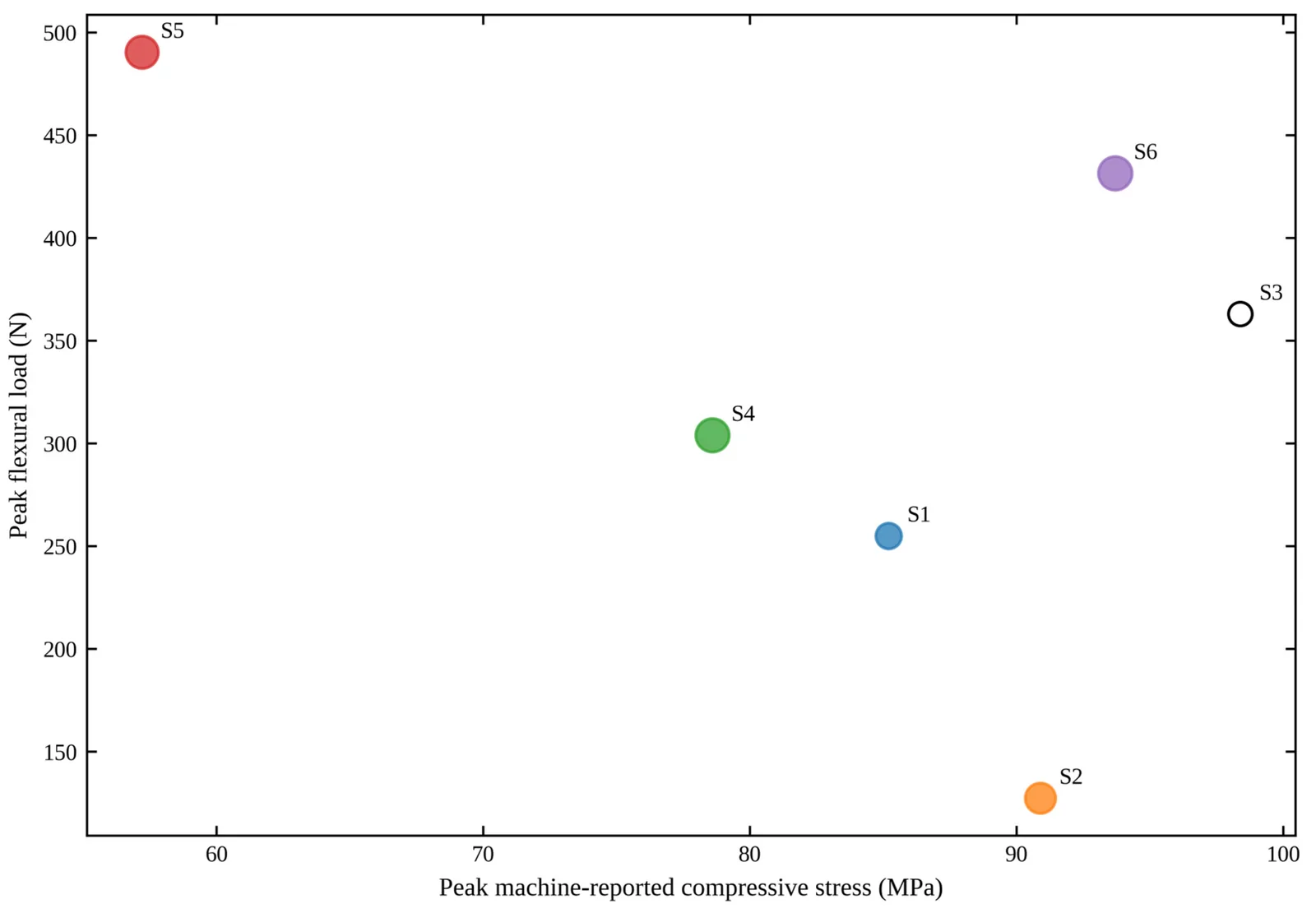
Cross-mode mechanical-performance map based on group-mean compression peak stress and flexural peak loads; marker size represents group-mean accepted tensile strength. Corresponding SD values are reported in Table 2. S3 is shown separately because its tensile result was rejected.

This distinction is captured by the cross-mode map in Figure 8 and the consolidated mean ± SD mechanical response tabulated in Table 2. The separation of S4, S5, and S6 illustrates loading-mode-dependent mechanical response states within this dataset rather than a single linear performance trajectory. In micromechanical terms, nHA primarily increases local stiffness, matrix constraint, and resistance to crack propagation, while Mg introduces a mechanically dissimilar phase that alters local compliance and stress redistribution. The relative fraction of these two phases may shift whether the response favors deformation accommodation, compressive confinement, or bending-load capacity [10,11,12,15,16,19].

### 3.2. Thermal Response by Differential Scanning Calorimetry

**Figure 9:**
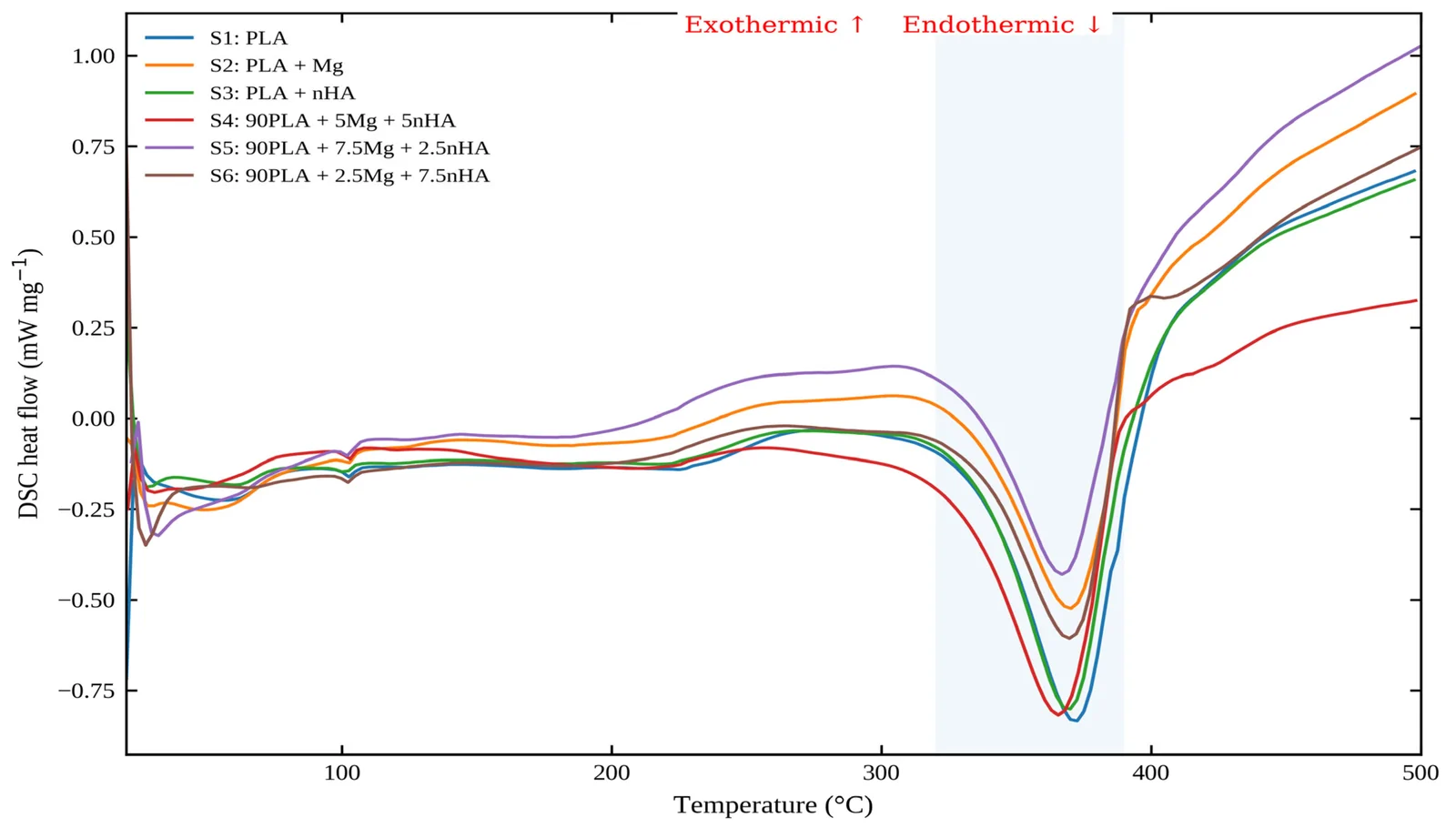
DSC heating traces of S1–S6 recorded from approximately 20 to 500 °C at 10 K min^−1^. The instrument convention is exothermic positive (upward) and endothermic negative (downward); the shaded interval contains the dominant high-temperature event used for comparative peak extraction.

**Figure 10:**
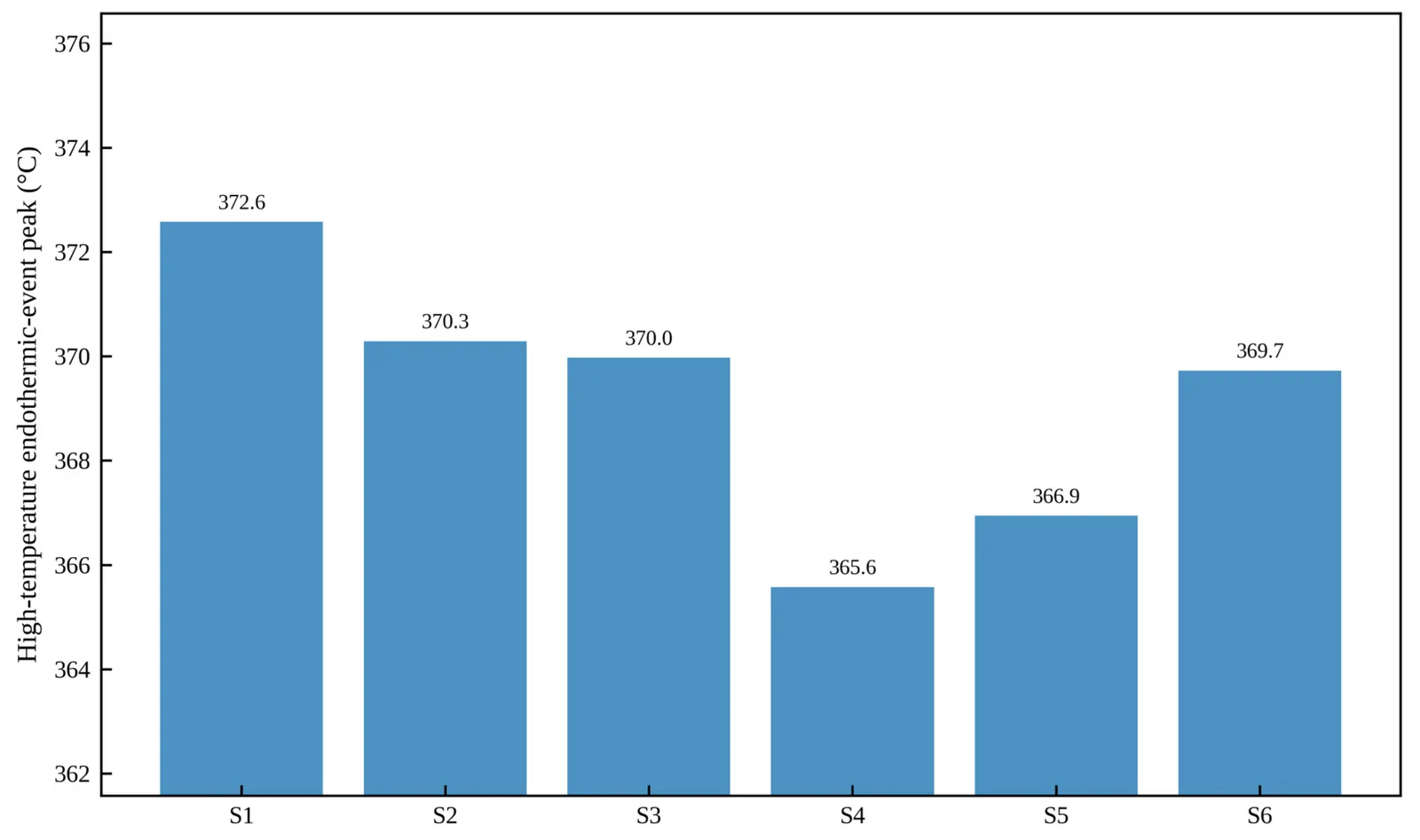
Peak temperature of the dominant high-temperature DSC event for the six formulations.

The DSC heating traces are compared in Figure 9, and the dominant high-temperature event is summarized in Figure 10 and Table 3. All compositions exhibit a pronounced high-temperature heat-flow event between approximately 365 and 373 °C under the specified purge conditions. The corresponding peak for neat PLA is 372.6 °C. The binary Mg and nHA composites show slightly lower values of 370.3 and 370.0 °C, respectively, while the ternary hybrids show stronger composition dependence: 365.6 °C for S4, 366.9 °C for S5 and 369.7 °C for S6. Relative to S1, the shifts are −7.0, −5.7 and −2.9 °C for the three hybrids. These values are operational DSC heat-flow event peaks and are not reported as conventional PLA melting temperatures or definitive decomposition temperatures. Because one archived DSC scan was available per composition, the inter-composition peak shifts are treated as descriptive single-run differences and are not assigned statistical significance.

The shift in this feature with composition shows that the recorded high-temperature heat-flow response changes among formulations. Because the scan extends to 500 °C and includes an oxygen purge stream, the signal is best regarded as an operational high-temperature DSC event under oxidative/mixed-purge conditions rather than as a conventional melting transition or a stand-alone decomposition temperature. In this temperature range, overlapping contributions from chain scission, volatilization, oxidation, and heat-capacity changes may occur, and DSC alone cannot separate or quantify them. The reported peak is therefore used only as a comparative heat-flow marker within the present test protocol; it is neither T_m_ nor T_d_, and determination of decomposition kinetics or thermal stability would require complementary TGA/DTG [5,18,19].

The comparatively small peak shifts in S2 and S3 are consistent with a smaller perturbation of the high-temperature heat-flow signature than in S4 and S5. The larger shift in S4 may reflect differences in filler distribution, thermal transport or the local oxidative/degradation environment created by the mixed metallic and ceramic phases. These possibilities are offered as qualitative interpretations only; the DSC data do not isolate a single reaction pathway.

The event temperature shifts back toward the neat-PLA value in S6, remaining only 2.9 °C below S1. This observation indicates that the nHA-rich formulation has a high-temperature heat-flow signature closer to neat PLA than S4 or S5 under the same scan conditions. It should not be interpreted as evidence of a higher melting point, a definitive decomposition temperature or improved thermal stability without complementary thermal-analysis data [8,9,10,11,18,19,22,25].

**Figure 11:**
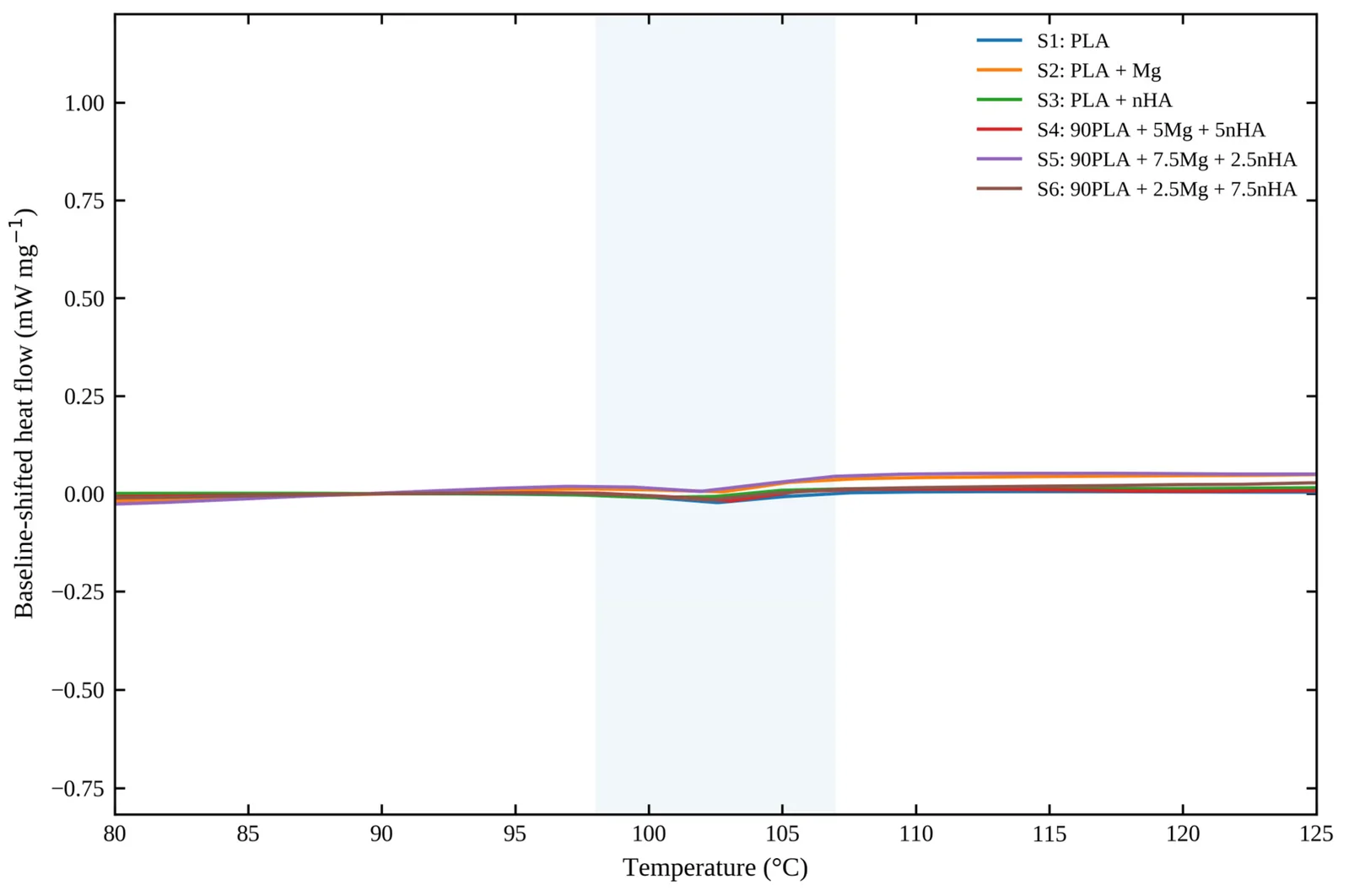
Expanded DSC response in the 80–125 °C region showing the low-amplitude transition-like feature near 100–105 °C.

A low-amplitude transition-like feature is present near 100–105 °C (Figure 11). Instrument analysis of S3 identified an onset at 101.7 °C, a midpoint at 102.9 °C, and an endpoint at 104.1 °C. The important observation is the persistence of a relatively small matrix-related relaxation feature rather than the formation of a new low-temperature transition in the hybrid series. Together with the XRD and FTIR results discussed below, the DSC response therefore indicates modification of the local matrix environment without evidence for wholesale replacement of the polymer phase [5,18].

A qualitative correspondence is observed between the composition-dependent mechanical response and the position of the high-temperature DSC event: S4 and S5 show the largest downward event shifts, whereas S6 remains closer to the neat-PLA value. This comparison is descriptive only. The DSC feature is an operational heat-flow marker under the specified oxidative/mixed-purge method and does not, by itself, establish a particular degradation pathway or interfacial mechanism.

### 3.3. X-Ray Diffraction: Phase-Sensitive Structural Response

**Figure 12:**
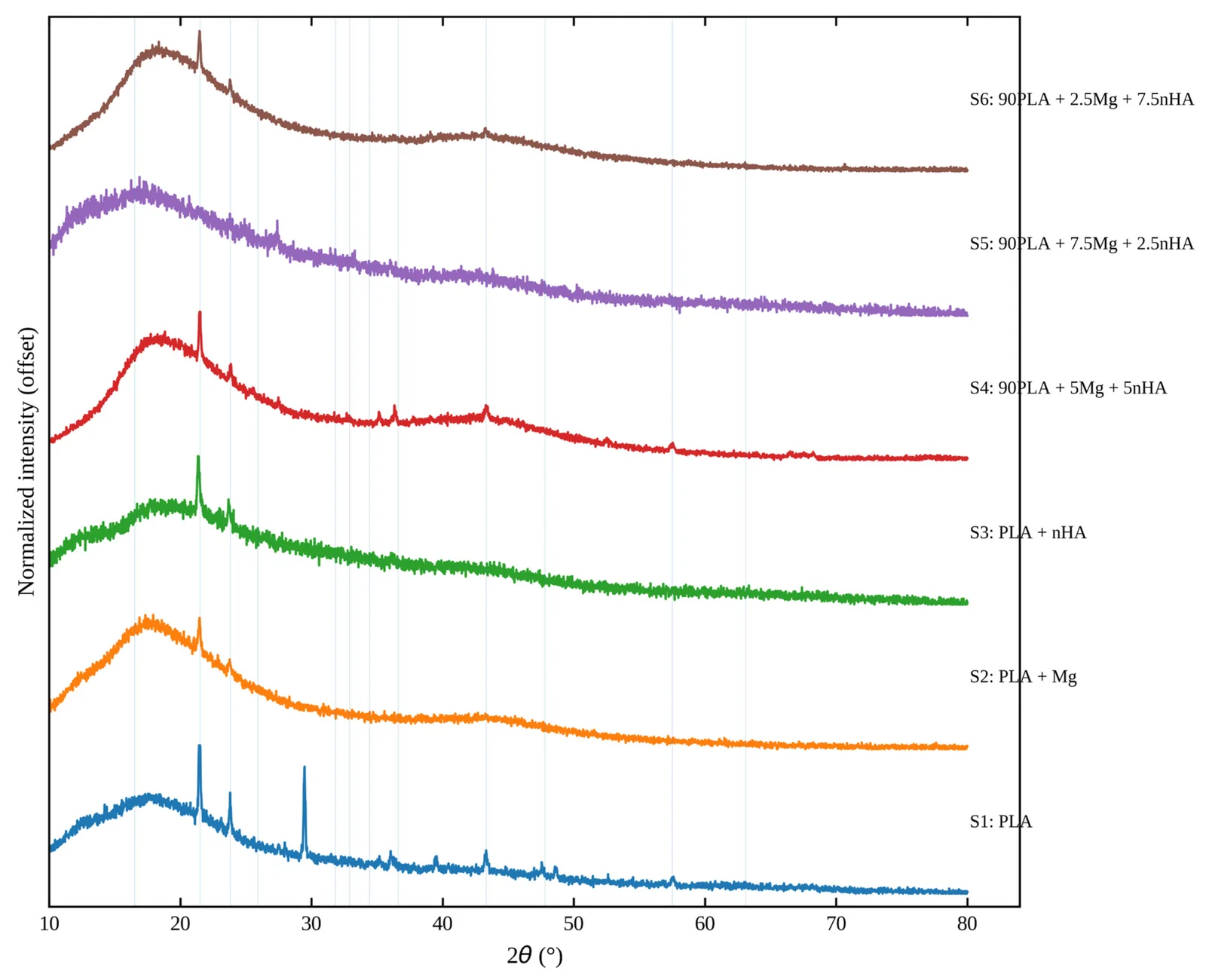
Normalized and vertically offset XRD patterns of S1–S6 showing the principal matrix-associated and reinforcement-sensitive diffraction regions.

The normalized and vertically offset XRD patterns are presented in Figure 12, and the principal detected features are summarized in Table 3. All compositions retain broad-to-semicrystalline diffraction features within approximately 2θ = 16–24°, with recurrent maxima in the regions of 16–17°, 21.4–21.5° and 23.7–23.9°. The persistence of these matrix-associated features indicates that the polymeric structural contribution remains dominant after incorporation of Mg and nHA [10,12,13,17,38].

Reinforcement changes the pattern primarily through the emergence and redistribution of narrower crystalline features superimposed on the polymer background rather than through replacement of the matrix pattern. S4 exhibits the clearest additional reflections, particularly near 36.37, 43.34, and 57.54°, whereas S5 shows comparatively weak diffraction contrast in the single archived scan and S6 retains a strongly matrix-dominated pattern with a visible feature near 43.27°. Several reflections of hydroxyapatite and hexagonal Mg occupy overlapping intermediate- and high-angle regions, so the composite patterns are most appropriately interpreted as evidence of retained crystalline reinforcement rather than uniquely separated phase fingerprints [13,17,38].

The weak S5 pattern is treated cautiously because the archived dataset contains no independent repeat XRD measurement for this formulation. The suppressed/absent reflections in that scan are therefore reported as an unverified feature of the single measurement and are not interpreted as proof that the corresponding crystalline phases are absent. No quantitative phase-fraction or crystallinity conclusion is based on the S5 intensity difference; repeat XRD measurements on independently prepared S5 specimens are required to establish reproducibility.

Within the available single-scan dataset, the absence of a new set of dominant reflections common to all three hybrids is chemically significant. Formation of a substantial new crystalline reaction product would be expected to generate reproducible new reflections across the hybrid compositions. Instead, the principal matrix features are preserved while the visibility of reinforcement-sensitive peaks changes with composition. The available patterns are therefore consistent with a multi-phase physical composite in which the starting constituents largely retain their structural identities [17,38].

The structural result is compatible with the mechanical interpretation but does not uniquely identify its cause. Large changes in strength and deformation occur without emergence of a new dominant bulk crystalline phase, suggesting that differences in phase distribution, matrix continuity, and particle–matrix interactions may contribute to the mechanical response. S4, for example, exhibits clear additional high-angle crystalline features, together with a high tensile stress and the largest tensile peak displacement ratio. This association should be viewed as correlative rather than as direct evidence of a specific interfacial stress-transfer mechanism. S5 shows a different structural–mechanical combination. Its diffraction contrast is weak, yet it produces the highest flexural peak load, indicating that the flexural response is not simply proportional to apparent bulk crystalline peak intensity. S6 is similarly matrix dominated in XRD but provides a broad mechanical response. These comparisons support the view that factors not resolved by bulk diffraction—including local particle distribution and interfacial conditions—may be important.

### 3.4. FTIR Spectroscopy: Molecular Structure and Interfacial Perturbation

**Figure 13:**
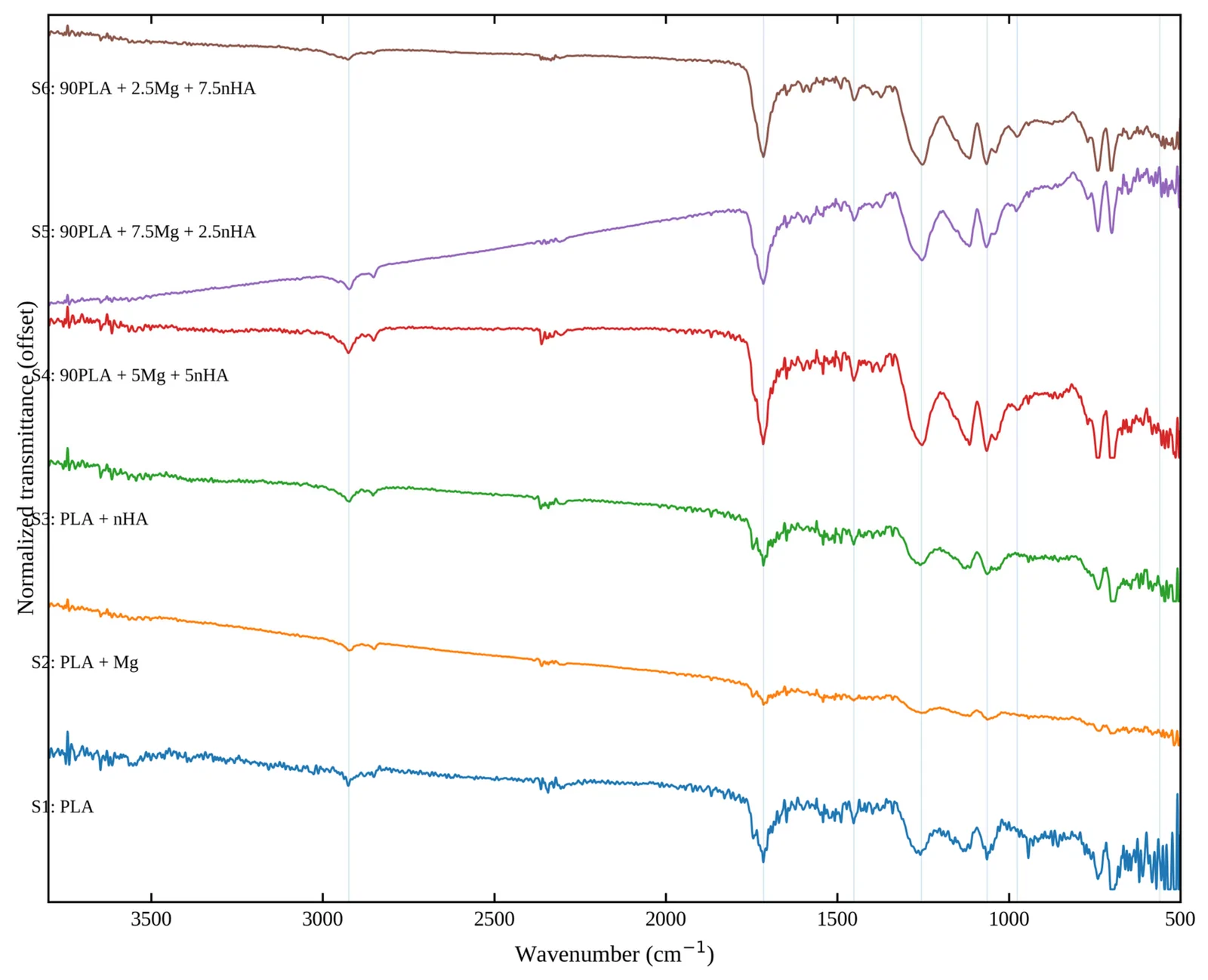
Normalized and vertically offset FTIR spectra of S1–S6 showing the principal matrix-associated and phosphate-sensitive spectral regions.

The normalized FTIR spectra are presented in Figure 13, and the selected band positions are summarized in Table 3. All compositions retain the principal vibrational framework of the polymer matrix. The dominant carbonyl-associated band remains at approximately 1715–1716 cm^−1^, C-H stretching occurs near 2922–2926 cm^−1^, the C-H bending region is centered near 1451–1452 cm^−1^, and strong C-O/C-O-C-associated features remain within approximately 1252–1258 and 1063–1066 cm^−1^. The persistence of these bands shows that reinforcement does not replace the dominant organic functional-group chemistry of the matrix [5,10,17,38].

The most systematic composition-dependent change occurs in the C-O/C-O-C region. The feature at 1258.3 cm^−1^ in S1 shifts to 1252.5 cm^−1^ in S4 and S5 and then to 1251.6 cm^−1^ in S6. A vibrational shift to a lower wavenumber reflects a change in the effective force constant and/or local dipole environment of the vibrating group. In the present system, the trend is consistent with progressive perturbation of ester/ether-containing polymer segments at heterogeneous particle interfaces [3,11,12].

The surface chemistry of nHA provides a physically plausible basis for this response. Hydroxyapatite surfaces contain Ca^2+^ sites, phosphate groups and surface hydroxyl species that can interact electrostatically or through hydrogen-bonding/dipolar interactions with polar groups of the polymer. Such interactions do not require formation of a new covalent bond to alter vibrational frequency; adsorption and local coordination can modify bond polarization and segmental mobility. The low-wavenumber displacement of the C-O/C-O-C region is therefore consistent with an increasingly perturbed interfacial environment in the ternary composites [3,11,12].

The hybrids also exhibit features near 975.8–978.7 cm^−1^ and within the 500–620 cm^−1^ region. These intervals are sensitive to phosphate-containing apatite vibrations and support retention of the nHA phase [1,3,4,7,8,9,38,39]. Elemental Mg, in contrast, does not produce a conventional molecular vibration in the mid-infrared region. Its influence on FTIR is therefore indirect, through surface chemistry and changes in local polymer packing rather than through the appearance of a discrete Mg-specific band.

The stability of the carbonyl-associated band is equally informative. S1, S2, S3, S5 and S6 retain the principal feature at approximately 1715.4 cm^−1^, while S4 shows only a small shift to 1716.3 cm^−1^. The absence of major new carbonyl, hydroxyl or phosphate-derived bands suggests that the processing route does not produce a new dominant organic chemistry. Together with the XRD results, this is consistent with a physically multi-phase composite and local interfacial perturbations; however, FTIR alone cannot establish interfacial bonding strength or a specific load-transfer mechanism [17,38,39].

The FTIR responses also show qualitative associations with mechanical behavior. S6 exhibits the largest displacement of the 1258 cm^−1^ feature to 1251.6 cm^−1^ while also showing high tensile, compressive, and flexural responses. This correspondence is consistent with a modified local chemical environment, but it should not be interpreted as proof that the FTIR shift causes the mechanical improvement. S4 and S5 show comparable shifts to 1252.5 cm^−1^ but with very different compressive and flexural behaviors, further indicating that the phase fraction, processing heterogeneity, and stress-state-dependent mechanics also contribute.

### 3.5. Integrated Composition–Structure–Property Relationship

When the mechanical, thermal, diffraction and spectroscopic results are considered together, a coherent but provisional composition–structure–property picture can be proposed, as indicated in Table 4. Varying the Mg:nHA ratio alters the relative fractions of the continuous PLA matrix, the calcium-phosphate reinforcement and the metallic Mg reinforcement. The measured properties therefore vary non-linearly, plausibly because these phases influence matrix mobility, stiffness contrast, local constraint and damage development in different ways. This interpretation is consistent with prior analyses of hydroxyapatite dispersion and polymer/ceramic interface quality [40,41], but the present study does not directly resolve particle-scale stress transfer or interfacial morphology.

S2 provides the clearest macroscopic baseline for the role of elemental Mg. Relative to neat PLA, it increases tensile stress by 69.4% and compressive stress by 6.7% but decreases flexural peak load by 50.0%. Its DSC event remains close to neat PLA at 370.3 °C, its XRD pattern remains predominantly matrix-like and its FTIR spectrum shows only a moderate C-O/C-O-C displacement from 1258.3 to 1254.5 cm^−1^. These observations are consistent with Mg-associated changes in mechanical response without a major change in the bulk chemical or crystalline framework. A stress-state-dependent interfacial explanation is plausible [15,17,18,19,36,42,43,44,45].

S3 provides the ceramic-dominated binary reference. Its tensile result was rejected by the acquisition system and is therefore excluded from tensile interpretation; however, it produces the highest mean compressive stress (98.4 ± 2.95 MPa) and a flexural peak load of 362.9 ± 10.89 N. The DSC event remains at 370.0 °C and the bulk XRD/FTIR framework remains matrix dominated. These results are consistent with the classical function of nHA as a rigid phase that increases local constraint and alters crack propagation without creating a new bulk polymer chemistry [1,3,4,7,8,9,10,11,12,13]. Comparable PLLA/HAp studies further show that ceramic reinforcement can raise stiffness and alter crack resistance while retaining a polymer-dominated composite framework, provided particle dispersion and interfacial bonding are adequately controlled [46,47].

S4 shows a pronounced mixed-composition response. It combines 52.0 ± 1.56 MPa tensile stress with the highest tensile peak displacement ratio (23.42 ± 0.70%), while its high-temperature DSC event occurs at the lowest value in the series (365.6 °C). It also exhibits clear additional high-angle XRD features and a C-O/C-O-C FTIR shift to 1252.5 cm^−1^. Taken together, these observations are consistent with substantial local matrix perturbation in the equal Mg:nHA composition; however, they do not directly establish the nature or strength of the particle–matrix interface.

S5 occupies a different regime. It produces the highest flexural peak load (490.4 ± 14.71 N) but the lowest compressive stress (57.2 ± 1.72 MPa). Its high-temperature DSC event occurs at 366.9 °C, the XRD pattern has weak overall diffraction contrast, and the C-O/C-O-C FTIR region remains shifted to 1252.5 cm^−1^. The bending response is therefore not explained by increased bulk crystallinity alone. A stress-field-specific redistribution mechanism is one plausible interpretation, but the absence of direct interfacial/fractographic evidence prevents a unique mechanistic assignment.

S6 shows a broad cross-mode macroscopic response within the recorded dataset. It provides the highest accepted mean tensile stress (52.8 ± 1.58 MPa), a high compressive stress (93.7 ± 2.81 MPa), and a high flexural peak load (431.5 ± 12.95 N). Its high-temperature DSC event at 369.7 °C is closer to neat PLA than those of S4 and S5, its XRD pattern remains matrix dominated, and its FTIR spectrum shows the largest low-wavenumber shift in the C-O/C-O-C feature to 1251.6 cm^−1^ together with phosphate-sensitive structure. This combination is compatible with a composition-dependent change in the local interfacial environment while the bulk matrix framework remains dominant, but it does not directly quantify interfacial coupling.

The difference between S5 and S6 is especially instructive at the macroscopic level. Increasing the Mg fraction from 2.5 to 7.5 wt.% raises the mean flexural peak load from 431.5 ± 12.95 to 490.4 ± 14.71 N, but simultaneously reduces the mean compressive stress from 93.7 ± 2.81 to 57.2 ± 1.72 MPa and the mean tensile stress from 52.8 ± 1.58 to 47.9 ± 1.44 MPa. Thus, changing the Mg:nHA partitioning is associated with a bending advantage for the Mg-rich composition and a more even balance of tensile, compressive, and flexural responses for the nHA-rich composition. Because total reinforcement remains fixed, this is a composition-partitioning trend. From a mechanical perspective, the data can be rationalized as a competition among matrix continuity, ceramic constraint and metallic stress redistribution. FTIR shows that the principal polymer functional groups are retained, while XRD remains matrix-dominated with composition-dependent reinforcement reflections. DSC provides an additional thermal-response signature; collectively, these datasets support a composition-dependent interphase hypothesis.

### 3.6. Comparison with Related PLA-HAp-Mg-Containing Systems

The magnitude and non-monotonicity of the present tensile response are consistent with the wider PLA-based composite literature. Injorhor et al. reported 38.21 MPa for neat PLA films, 54.45 MPa at 2.5 phr nHAp and 66.41 MPa at 5 phr, followed by a reduction to 45.80 MPa at 10 phr [8]. The accepted group-mean tensile values of 47.9 ± 1.44 to 52.8 ± 1.58 MPa for S4–S6 therefore fall within a physically credible range for particle-reinforced PLA systems and reproduce the broader observation that an intermediate reinforcement condition can outperform both the neat matrix and a higher single-filler fraction [8,10,11,12,13]. More recent PLA/nHA investigations likewise demonstrate that reinforcement level, printing/fabrication route, particle distribution and interfacial state jointly govern tensile, compressive, thermal and biological response rather than producing a simple filler-content trend [48,49,50,51,52,53].

A related study reported composition-dependent tensile and thermal behaviors following the combined addition of HAp and Mg(OH)_2_ [22]. The present system differs fundamentally because Mg is introduced as elemental metal rather than MgO, Mg(OH)_2_ or Mg substituted within the HAp lattice. The interface therefore contains a true polymer-metal component in addition to a polymer–ceramic interface, creating a broader distribution of local stiffness and surface chemistry [15,17,18,19].

Compressive behavior is particularly sensitive to the material architecture. Wang et al. reported a reduction in compressive strength after introducing a very high nHA fraction into porous printed PLA scaffolds [7], whereas Xu et al. observed increased yield stress following nHA and nMgO addition [21]. Evlen and Eroglu showed that PLA/HA/TiO2 compressive behavior changes substantially with the reinforcement ratio and porous structure [25]. These contrasting values reinforce the mechanistic interpretation adopted here, namely that ceramic and Mg-bearing phases do not produce a universal monotonic strengthening effect; the response depends on matrix continuity, porosity, filler fraction, interface condition and loading path [10,15,16].

Mg-containing HAp/PLA systems provide complementary chemical context. Mg-doped nHA has been shown to influence osteogenic response in PLA-based scaffolds [20], while Mg-Zn-substituted HAp/PLA systems exhibit altered interfacial, structural and biological behavior [23,24]. Hybrid reinforcement effects have also been reported in Mg-based HAp composites [26]. Although these systems differ from the discrete elemental-Mg/nHA formulation investigated here, they collectively establish that Mg-bearing and apatite phases can interact non-additively with their surrounding matrix [3,15,16,17,18]. Related Mg/PLA and PLA-Mg(OH)_2_-nHA studies also show that the mechanical benefit of Mg-containing phases is inseparable from interfacial design and degradation behavior, reinforcing the need to evaluate composition as a coupled mechanical-physicochemical variable [54,55].

The distinguishing contribution of the present work is therefore the direct comparison of discrete elemental Mg and nHA at a fixed total reinforcement fraction. The results show that Mg:nHA partitioning controls not only the magnitude of the mechanical response but also the loading mode in which that response is expressed. This transforms the composition variable from a simple filler percentage into a design parameter for selecting between tension-dominant, flexure-dominant and mechanically balanced material states.

### 3.7. Mechanistic Synthesis and Materials-Design Implications

The combined results can be rationalized using a three-phase framework in which the continuous PLA matrix provides the baseline deformation capacity, nHA may act as a relatively rigid constraining phase, and Mg may act as a mechanically dissimilar stress-redistribution phase. This framework is useful for interpreting the loading-mode dependence observed here and is consistent with previous composite studies [3,10,11,12,13,15,16,19].

At low or balanced Mg fractions, the measured tensile response is consistent with stress redistribution that does not excessively disrupt matrix continuity. When Mg becomes dominant within the fixed 10 wt.% filler budget, as in S5, the material shows a high bending capacity but lower compressive resistance. Conversely, the nHA-rich S6 exhibits a more uniform macroscopic response across loading modes. These observations are compatible with changes in local compliance, matrix constraints, and shear transfer [10,11,12,15,16,19].

The thermal and spectroscopic data provide supporting but indirect context for this framework. XRD retains matrix-dominated diffraction features and FTIR retains the principal organic bands, while the C-O/C-O-C region shifts with the composition. These observations are consistent with local changes in the polymer–particle environment without evidence for a new dominant bulk reaction phase [3,17,38,39]. Within this provisional framework, S6 shows a broad combination of tensile, compressive, and flexural responses in the investigated composition window, while S5 is associated with the highest mean flexural load and S4 with the greatest mean tensile peak displacement. The mechanical data are supported by n = 10 replicate statistics, with relatively small within-group SDs; however, because inferential significance testing and independent batch-to-batch replication were not undertaken, the present dataset does not establish statistical superiority of any formulation [56].

### 3.8. Study Limitations

This study was intentionally limited to one total reinforcement level (10 wt.%) and three ternary Mg:nHA ratios (1:1, 3:1, and 1:3), together with binary controls. The observations therefore define composition-response trends only within this experimental window and do not establish a global optimum, nor do they predict behaviors at other total filler contents, particle characteristics, processing routes, or specimen geometries.

Mechanical replication comprised n = 10 specimens per formulation per loading mode (five initial and five confirmatory specimens), and the ten specimen-level peak measurements were consolidated as mean ± SD. The resulting SDs provide a direct measure of within-series repeatability and are approximately 2.9–3.0% of the corresponding means for the accepted mechanical metrics. Nevertheless, these specimens represent repeat tests within the present experimental series rather than independent batch-to-batch replication, and no ANOVA or pairwise inferential significance testing was performed. The mechanical comparisons should therefore be interpreted as descriptive composition-dependent differences supported by within-group variability estimates rather than as formal claims of statistically significant superiority. The S3 tensile result remains excluded from tensile-performance interpretation because of its rejected acquisition-system status.

Physicochemical characterization is also limited by replication and method scope: one archived DSC scan, one XRD pattern, and one FTIR spectrum were available per formulation. The weak S5 XRD pattern was not independently repeated; DSC alone cannot establish decomposition kinetics or thermal stability; and no direct SEM/fractography, particle-dispersion, porosity, or interface characterization was available to verify the proposed particle-scale mechanisms. Finally, the biological relevance of PLA, nHA, and Mg is based on constituent selection and the contextual literature rather than direct degradation, ion-release, bioactivity, cytocompatibility or in vivo measurements. Future work should combine independently prepared batches, complete specimen-level statistical analysis, repeat DSC/XRD/FTIR, complementary TGA/DTG, quantitative microscopy/porosity analysis, and biological testing across a broader composition space.

## 4. Conclusions

At a fixed 10 wt.% total reinforcement content, changing the Mg:nHA partitioning produced loading-mode-dependent mechanical and physicochemical responses rather than a monotonic strengthening trend. The mechanical results were consolidated from n = 10 specimens per formulation and loading mode and are reported as mean ± SD, with relatively small within-group dispersion for the accepted metrics. Within this dataset, the 1:1 hybrid combined a high tensile stress with the largest tensile peak displacement, the Mg-rich 3:1 hybrid showed the largest flexural peak load, and the nHA-rich 1:3 hybrid retained a broader combination of tensile, compressive and flexural responses. These observations identify reinforcement partitioning as a useful composition variable within the tested formulation window.

The thermal and structural measurements support a composition-dependent but matrix-dominated response. The high-temperature DSC feature occurred between 365.6 and 372.6 °C under the specified mixed-purge conditions and is treated only as an operational heat-flow marker. XRD retained the principal matrix-associated pattern with composition-sensitive reinforcement features, while FTIR preserved the main PLA bands and showed a systematic C-O/C-O-C shift in the ternary hybrids. These observations are consistent with changes in the local polymer–particle environment, but they do not directly prove a particular interfacial load-transfer mechanism.

Mechanical testing comprised n = 10 specimens per formulation per loading mode, and the specimen-level peak measurements were consolidated as arithmetic mean ± SD. The small within-group SDs support good repeatability of the accepted mechanical peak responses within the present test series. However, no inferential significance testing or independent batch-to-batch replication was undertaken, so the conclusions are framed in terms of composition-response trends rather than statistically significant superiority. DSC, XRD, and FTIR were represented by one archived measurement per formulation and therefore remain descriptive. Broader composition screening, independently prepared replicate batches, inferential mechanical statistics, repeat structural/thermal characterizations, direct microstructural analyses, and degradation/cytocompatibility studies are required before biomedical performance or an optimal formulation can be established.

## Figures and Tables

**Table 1:** Composition matrix and specimen coding used for the PLA-nHA-Mg composite system. All compositions are expressed as wt.% of the total composite mass.

Code	Designation	Archived DSC Code	PLA (wt.%)	Mg (wt.%)	nHA (wt.%)	Total Reinforcement (wt.%)	Mg:nHA Ratio	Design Role
S1	PLA	PLA	100.0	0	0	0	-	Neat PLA reference
S2	PLA-10Mg	PLA + Mg	90.0	10.0	0	10.0	Mg only	Binary Mg control
S3	PLA-10nHA	PLA + HA	90.0	0	10.0	10.0	nHA only	Binary nHA control
S4	PLA-5Mg-5nHA	C1	90.0	5.0	5.0	10.0	1:1	Balanced hybrid
S5	PLA-7.5Mg-2.5nHA	C2	90.0	7.5	2.5	10.0	3:1	Mg-rich hybrid
S6	PLA-2.5Mg-7.5nHA	C3	90.0	2.5	7.5	10.0	1:3	nHA-rich hybrid

**Table 2:** Consolidated mechanical response of neat PLA and PLA-Mg-nHA composites. Ten specimens were tested per formulation per mechanical assay (five initial and five confirmatory specimens). For each mechanical parameter, the ten specimen-level peak values were consolidated as arithmetic mean ± sample SD (n = 10). The S3 tensile result was classified as rejected by the acquisition system and is retained only for traceability; it is excluded from tensile-performance ranking.

Sample	Tensile Stress (MPa)	Tensile Peak Disp. (%)	Compression Stress (MPa)	Compression Peak Disp. (%)	Flexural Peak Load (N)	Flexural Nominal Stress (MPa)	Status
S1	23.5 ± 0.70	9.92 ± 0.30	85.2 ± 2.56	66.0 ± 1.98	255.0 ± 7.65	4.0 ± 0.12	Accepted
S2	39.8 ± 1.19	13.70 ± 0.41	90.9 ± 2.73	56.4 ± 1.69	127.5 ± 3.82	2.0 ± 0.06	Accepted
S3	0.7 ± 0.02 *	0.24 ± 0.01 *	98.4 ± 2.95	113.2 ± 3.40	362.9 ± 10.89	4.8 ± 0.14	Tensile rejected
S4	52.0 ± 1.56	23.42 ± 0.70	78.6 ± 2.36	56.0 ± 1.68	304.0 ± 9.12	4.0 ± 0.12	Accepted
S5	47.9 ± 1.44	21.00 ± 0.63	57.2 ± 1.72	47.6 ± 1.43	490.4 ± 14.71	6.4 ± 0.19	Accepted
S6	52.8 ± 1.58	18.82 ± 0.56	93.7 ± 2.81	92.0 ± 2.76	431.5 ± 12.95	5.7 ± 0.17	Accepted

* Rejected tensile result; mean ± SD retained only for traceability and excluded from tensile-performance interpretation.

**Table 3:** Consolidated physicochemical summary of the six formulations. DSC, XRD, and FTIR entries are derived from one archived analytical measurement per formulation and are interpreted descriptively rather than as group means.

Sample	DSC Event Peak (°C)	XRD Prominent Features, 2θ (°)	XRD Interpretation	FTIR C-O/C-O-C Band Position (cm^−1^)	FTIR Phosphate-Sensitive Features (cm^−1^)
S1	372.6	21.47, 29.48, 23.80, 43.32, 36.04, 39.49	Matrix-dominated pattern; narrow features retained	1258.3	945.9; 514.9
S2	370.3	21.45, 17.19, 23.80	Matrix response with limited additional crystalline contrast	1254.5	945.9; 503.3
S3	370.0	21.38, 23.69	Low-contrast matrix-dominated pattern	1258.3	945.9; 503.3
S4	365.6	21.49, 23.86, 17.34, 43.34, 36.37, 57.54	Most pronounced additional high-angle crystalline features	1252.5	975.8; 513.9
S5	366.9	27.39	Weak contrast in single archived scan; not independently confirmed	1252.5	978.7; 503.3
S6	369.7	21.47, 17.11, 23.82, 43.27	Matrix-dominated pattern with retained high-angle feature	1251.6	975.8; 518.8

**Table 4:** Integrated composition–structure–property interpretation of the PLA-Mg-nHA system.

Sample	Dominant Mechanical Response	DSC Response	XRD/FTIR Response	Integrated Interpretation
S1	Baseline matrix response	372.6 °C	Matrix reference	Continuous polymer reference
S2	Improved tensile; low flexural load	370.3 °C	Minor FTIR perturbation; matrix-dominated XRD	Higher tensile response associated with Mg addition; interfacial mechanism proposed but not directly observed
S3	Highest compression; improved flexure	370.0 °C	Matrix-dominated pattern with nHA-sensitive contribution	Compression/bending response consistent with ceramic constraint; local mechanism not directly observed
S4	High tensile stress; highest tensile displacement	365.6 °C	Strongest additional XRD features; C-O/C-O-C shift	Balanced hybrid with tension-favored macroscopic response; mixed-interface interpretation is provisional
S5	Highest flexural load; lowest compression	366.9 °C	Weak XRD contrast in single archived scan (not independently confirmed); C-O/C-O-C shift	Mg-rich, bending-dominant recorded response; particle-scale mechanism unresolved
S6	Highest accepted tensile, with high compression and flexure	369.7 °C	Matrix-dominated XRD; largest C-O/C-O-C shift; phosphate-sensitive bands	nHA-rich, broad cross-mode recorded response; interphase/load-transfer mechanism proposed rather than directly measured

## Data Availability

Data supporting the findings of this study are contained within the article. Additional data are available from the corresponding authors upon reasonable request.
